# The Gut Microbiota Affects Anti‐TNF Responsiveness by Activating the NAD^+^ Salvage Pathway in Ulcerative Colitis

**DOI:** 10.1002/advs.202413128

**Published:** 2024-12-30

**Authors:** Jing Lei, Lin Lv, Li Zhong, Feng Xu, Wenhao Su, Yan Chen, Zhixuan Wu, Song He, Yongyu Chen

**Affiliations:** ^1^ Department of Gastroenterology The Second Affiliated Hospital of Chongqing Medical University Chongqing 400010 China; ^2^ Department of Gastroenterology Renmin Hospital of Wuhan University Hubei 430060 China; ^3^ Department of Dermatovenereology Chengdu Second People's Hospital Sichuan 610011 China

**Keywords:** anti‐tumor necrosis factor therapy, *fusobacterium nucleatum*, microbiota, NAD^+^ salvage pathway, ulcerative colitis

## Abstract

Approximately 50% of the patients with ulcerative colitis (UC) are primarily nonresponsive to anti‐tumor necrosis factor (TNF) therapy or lose their responsiveness over time. The gut microbiota plays an important role in the resistance of UC to anti‐TNF therapy; however, the underlying mechanism remains unknown. Here, it is found that the transplantation of gut fecal microbiota from patients with UC alters the diversity of the gut microbiota in dextran sulfate sodium‐induced colitis mice and may affect the therapeutic responsiveness of mice to infliximab. Furthermore, the abundances of *Romboutsia* and *Fusobacterium* increase in the tissues of patients with UC who do not respond to anti‐TNF therapy. Differentially abundant metabolites are mainly enriched in nicotinate and nicotinamide metabolism in NCM460 cells after *Fusobacterium nucleatum* infection. Mechanistically, *F. nucleatum* promotes the nicotinamide adenine dinucleotide (NAD^+^) salvage pathway by upregulating NAMPT expression, which subsequently leads to the activation of the p38 mitogen‐activated protein kinase (MAPK) signaling pathway and promotes the secretion of inflammatory factors, ultimately inhibiting the therapeutic response to anti‐TNF drugs. These findings demonstrate that the gut microbiota can influence the response to anti‐TNF therapy in patients with UC and highlight the therapeutic potential of targeting *F. nucleatum* and its associated pathways for preventing and treating drug resistance in UC.

## Graphical abstract

The gut microbiota affects the response to anti‐TNF therapy in UC patients. *F. nucleatum* activates the p38 MAPK signaling pathway by regulating the NAD^+^ salvage pathway to inhibit anti‐TNF therapy responsiveness. Interfering with the NAD^+^ salvage pathway or inhibiting the growth of *F. nucleatum* may alleviate the resistance of UC to anti‐TNF therapy.

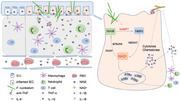

## 1 Introduction

Ulcerative colitis (UC), a major form of inflammatory bowel disease (IBD), is a chronic inflammatory disorder of unknown etiology with a rapidly increasing incidence in developing countries.^[^ ^1^ ^]^ Anti‐tumor necrosis factor (TNF) therapy is the primary treatment for moderate‐to‐severe UC; however, approximately half of patients with UC are primary non‐responders to anti‐TNF therapy, and ≈40% of patients who initially respond to treatment may lose their responsiveness over time.^[^ ^2^ ^]^ Therefore, exploring the mechanisms of anti‐TNF therapy resistance in UC is essential for optimizing current therapeutic strategies.

Recent studies have suggested that the gut microbiota plays an important role in the response to anti‐TNF therapy.^[^ ^2^, ^3^, ^4^, ^5^, ^6^, ^7^ ^]^ Baseline microbial richness analyses indicate a preferential response to anticytokine therapy.^[^ ^5^ ^]^ A high absolute abundance of *Bifidobacteriales* in the gut microbiota of pediatric patients with IBD is associated with the response to infliximab (IFX) treatment.^[^ ^6^ ^]^ Recently, a study using 16S rRNA sequencing of fecal samples from patients with IBD, including those from patients with UC and patients with Crohn's disease (CD), revealed that non‐responders had a lower abundance of short‐chain fatty acid producers, particularly those of the class *Clostridia*, and a higher abundance of pro‐inflammatory bacteria and fungi, such as those of the genus *Candida*, than responders.^[^ ^2^ ^]^ Another 16S rDNA sequencing study of CD tissues revealed that the abundance of *Fusobacteria* was higher in the feces of patients who did not respond to anti‐TNF therapy than in those who did respond,^[^ ^7^ ^]^ suggesting that *Fusobacteria* may be related to CD anti‐TNF responsiveness. *Fusobacterium nucleatum* (*F. nucleatum*) infection can exacerbate UC. The abundance of *F. nucleatum* in UC tissues was found to be higher than in normal tissues.^[^ ^8^, ^9^, ^10^, ^11^ ^]^ *F. nucleatum* promotes intestinal epithelial cell autophagy,^[^ ^9^ ^]^ regulates M1 macrophage polarization,^[^ ^11^ ^]^ or activates IL‐17F signaling^[^ ^8^ ^]^ to aggravate intestinal barrier damage and colonic inflammation in UC, suggesting *F. nucleatum* can induce inflammation and immune responses in UC. However, there are currently no specific biological markers to evaluate the responsiveness of UC to anti‐TNF therapy. Moreover, the relationship between the microbiome and the anti‐TNF response in UC and its possible molecular mechanisms have not yet been investigated.

Nicotinamide adenine dinucleotide (NAD^+^) is an important coenzyme in biological metabolism involved in oxidative phosphorylation, DNA repair, and inflammation.^[^ ^12^ ^]^ The expression of nicotinamide phosphoribosyl transferase (NAMPT), a rate‐limiting enzyme of the NAD^+^ salvage pathway, is elevated in the intestinal tissues of children with IBD and may serve as a marker of disease severity in pediatric patients with IBD.^[^ ^13^ ^]^ The NAMPT inhibitor FK866 potently blocks the NAD^+^ salvage pathway, reduces intestinal mucosal NAD^+^ levels, reduces the abundance and activity of NAD‐dependent enzymes, including PARP1, Sirt6, and CD38, reduces nuclear factor‐kappa B activation, and decreases cellular infiltration by inflammatory monocytes, macrophages, and activated T cells, thereby inhibiting the occurrence of colitis and colitis‐related tumors in mice.^[^ ^14^ ^]^ Various metabolic biomarkers involving lipid, bile acid, and amino acid pathways may contribute to the prediction of the response to anti‐TNF therapy in patients with IBD;^[^ ^15^ ^]^ however, the correlation between NAD^+^ metabolism and anti‐TNF therapy responsiveness in patients with UC remains unclear. One study showed that the serum levels of NAMPT are increased in patients with IBD who fail to respond to anti‐TNF therapy and decreased in patients who are responsive to these therapies.^[^ ^16^ ^]^ A study on anti‐TNF therapy for UC showed that NAMPT could be used to predict drug non‐responsiveness.^[^ ^17^ ^]^ However, whether NAMPT affects the efficacy of UC anti‐TNF therapy by regulating the NAD^+^ salvage pathway and the correlation between the gut microbiota and NAD^+^ metabolism has not yet been elucidated.

Mitogen‐activated protein kinase (MAPK) is a key regulatory pathway for cell proliferation, differentiation, transformation, and apoptosis. The MAPK signaling pathway is associated with the responsiveness to anti‐TNF therapy.^[^ ^18^, ^19^ ^]^ A previous study has shown that p38δ protein levels are significantly increased in the synovial tissue of rheumatoid arthritis patients who do not respond to infliximab; further studies on single nucleotide polymorphisms (SNPs) in the MAPK pathway revealed that both upstream and downstream SNPs of the p38MAPK cascade are associated with anti‐TNF therapy responsiveness.^[^ ^18^ ^]^ Alterations in p‐p38 and p‐JNK levels in the peripheral blood of IFX‐treated patients with bipolar depression are strongly associated with the remission of depressive symptoms.^[^ ^19^ ^]^ A previous study has shown that NAMPT promotes the phosphorylation of p38MAPK.^[^ ^20^ ^]^ The exoprotein Gbp of *F. nucleatum* promotes THP‐1 cell lipid deposition by binding to CypA and activating the PI3K‐AKT/MAPK/NF‐kappaB pathways.^[^ ^21^ ^]^ However, whether the gut microbiota regulates anti‐TNF responsiveness via p38MAPK during anti‐TNF therapy in UC remains unclear.

Here, we explored whether and how the gut microbiota affects the response to anti‐TNF therapy in patients with UC. We found that alterations in the gut microbiota were associated with the response to anti‐TNF therapy in patients with UC and that the abundance of *F. nucleatum* was significantly increased in patients with UC who did not respond to anti‐TNF therapy. We also demonstrated that *F. nucleatum* inhibited the responsiveness of UC to anti‐TNF therapy by increasing NAMPT expression, modulating the NAD^+^ salvage pathway, and activating the p38 MAPK signaling pathway.

## 2 Results and Discussion

### 2.1 The Gut Microbiota Affects the Therapeutic Response of DSS‐Induced Colitis to IFX

**Figure 1:**
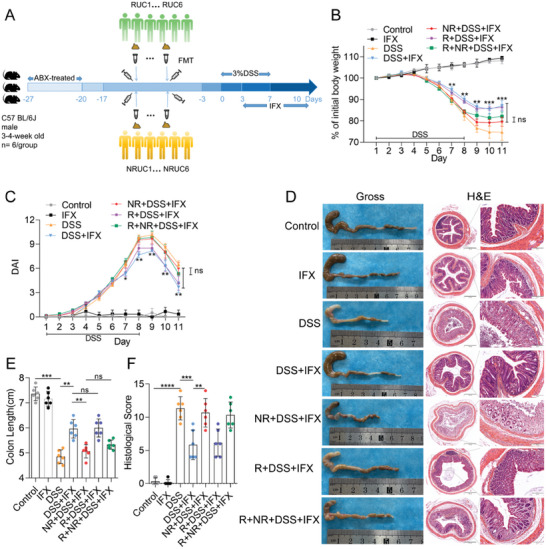
Gut microbiota affects the response of DSS‐induced colitis to IFX therapy. A) Schematic representation of experimental setup for mice. B,C) Statistical analysis of body weight change B) and DAI C) of mice (*n* = 6 per group; ns = no significance, *p < 0.05, **p < 0.01, and ***p < 0.001; the dividing lines indicate any joins; one‐way ANOVA combined with Bonferroni's *post hoc* test. Error bars indicate SD). D) Representative image of colons and H&E staining of colon sections from mice of each group (4 × magnification and 200 × magnification). E,F) Statistical analysis of colon length E) and histopathological score of the colon F) (*n* = 6 per group; ns = no significance, *p < 0.05, **p < 0.01, and ***p < 0.001; the dividing lines indicate any joins; nonparametric Mann‐Whitney U test. Error bars indicate SD).

To investigate the potential relationship between alterations in the gut microbiota and the therapeutic effect of anti‐TNF drugs in the treatment of UC, we performed fecal microbiota transplantation (FMT) in broad‐spectrum antibiotic (ABX) model mice using feces from patients with UC who responded to anti‐TNF therapy (RUC) and patients with UC who did not respond to anti‐TNF therapy (NRUC) (**Figure** **1**A). Compared with mice co‐treated with dextran sodium sulfate (DSS) and IFX, mice transplanted with fecal microbiota from NRUC patients (NR+DSS+IFX) presented rapid weight loss (*p <* 0.01; Figure 1B), higher disease activity index (DAI) (*p <* 0.05; Figure 1C), more severe disease and disruption of mucosal structures (Figure 1D), shorter colon lengths (*p* < 0.01; Figure 1E) and higher histological scores (HS) (*p* < 0.01; Figure 1F). These data suggest that gut microbiota transplantation from NRUC patients may affect the response to IFX treatment in mice with DSS‐induced colitis. Additionally, we detected a more significant weight loss (*p* < 0.01; Figure 1B), higher DAI (*p* < 0.01; Figure 1C), more severe disease and disruption of mucosal structures (Figure 1D), shorter colon length (*p* < 0.01; Figure 1E), and a significant increase in HS (*p* < 0.01; Figure 1F) in the NR+DSS+IFX group than in mice co‐treated with DSS and IFX after transplantation with fecal microbiota from RUC patients (R+DSS+IFX). These findings indicate that the transplantation of gut fecal microbiota from UC patients of different origins leads to different responses to IFX treatment in mice with DSS‐induced colitis.

To further investigate whether transplanting fecal microbiota from RUC patients could alleviate the drug resistance caused by transplanting fecal microbiota from NRUC patients, we simultaneously transplanted fecal microbiota from RUC and NRUC patients (R+NR+DSS+IFX). Compared with the NR+DSS+IFX group, the weights of the mice in the R+NR+DSS+IFX group were not significantly lower (*p* > 0.05; Figure 1B), the DAI was not significantly lower (*p* > 0.05; Figure 1C), the severity of the disease and destruction of the mucosal structure were not significantly different (Figure 1D), and the differences in colon length (*p* > 0.05; Figure 1E) and HS were not significant (*p* > 0.05; Figure 1F). These results suggest that simultaneous transplantation of fecal microbiota from RUC and NRUC patients did not affect the responsiveness of mice with DSS‐induced colitis to IFX treatment. Collectively, our data indicate that the fecal microbiota of patients with UC may affect the therapeutic responsiveness of DSS‐induced colitis to IFX.

### 2.2 FMT Alters the Diversity of the Gut Microbiota in Mice with DSS‐Induced Colitis

To analyze the bacterial composition in the intestinal tissues of each group of mice, we compared the sequencing data generated using the Illumina NovaSeq 6000 from DSS+IFX, NR+DSS+IFX, R+DSS+IFX, and R+NR+DSS+IFX mouse tissues. The alpha diversity was then analyzed for each group. The Chao1 index of the NR+DSS+IFX group was significantly higher than that of the DSS+IFX group (*p* < 0.01; Figure S1, Supporting Information). Moreover, the Simpson and Shannon indices were significantly higher in the NR+DSS+IFX group than in the R+DSS+IFX group (*p* < 0.01; Figure S1E,F, Supporting Information). However, there was no significant difference in alpha diversity between the NR+DSS+IFX and NR+R+DSS+IFX groups (*p* > 0.05; Figure S1G–I, Supporting Information).

**Figure 2:**
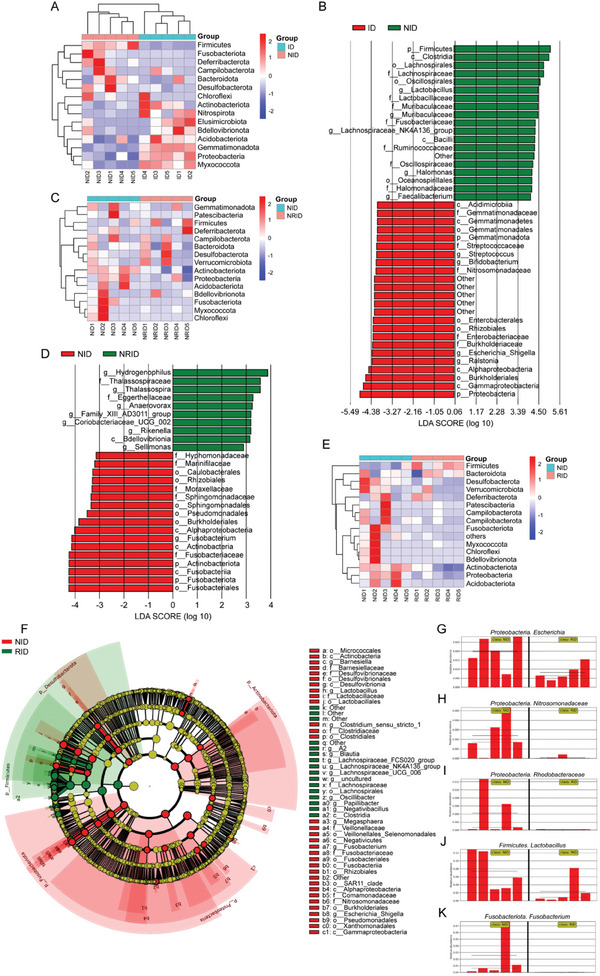
Changes of microbial diversity in intestinal tissues of mice with DSS‐induced colitis. A, C, E) Hierarchically clustered heat map analysis of the highly represented bacterial taxa (phylum level) in tissues from ID (DSS+IFX) and NID (NRUC+DSS+IFX) mice A), NID and NRID (NRUC+RUC+DSS+IFX) mice C), NID and RID (RUC+DSS+IFX) mice E) by 16S rDNA sequencing. B, D) LEfSe analysis identifies the relative taxa abundance from ID (red) and NID (green) mice B), NID (red), and NRID (green) mice D). Only taxa with values greater than the LDA threshold of 3.6 are shown. F) A cladogram representation of data from NID (red) and RID (green) mice by 16S rDNA sequencing. G–K) The abundance of *Escherichia* G), *Nitrosomonadaceae* H), Rhodobacteraceae I), *Lactobacillus* J), *Fusobacterium* K) from NID and RID tissues by 16S rDNA sequencing.

We further evaluated the β diversity of the gut microbiota in the intestinal tissues of each group. Compared with those in the DSS+IFX group, the abundances of *Firmicutes*, *Fusobacteriota*, *Deferribacterota*, *Campilobacterota* and *Bacteroidota* in the tissues of the NR+DSS+IFX group were significantly higher, whereas the abundances of *Myxococcota*, *Proteobacteria*, *Gemmatimonadota*, *Acidobacteriota* and *Actinobacteriota* were significantly lower (*p* < 0.05; **Figure** **2**A). *Clostridia*, *Lachnospirales*, *Lactobacillus*, and *Fusobacteriaceae* were abundant in the NR+DSS+IFX group, whereas *Proteobacteria*, *Ralstonia*, *Bifidobacterium*, and *Streptococcus* were abundant in the DSS+IFX group (*p* < 0.05; Figure 2B). Compared with those in the NR+DSS+IFX group, the abundances of *Firmicutes*, *Desulfobacterota*, *Bacteroidota*, and *Verrucomicrobiota* in the NR+R+DSS+IFX group were significantly higher, whereas the abundances of *Actinobacteriota*, *Proteobacteria*, *Acidobacteriota*, and *Fusobacteriota* were lower (*p* < 0.05; Figure 2C). *Hydrogenophilus*, *Thalassospira*, *Anaerovorax*, and *Rikenella* were abundant in the NR+R+DSS+IFX group, whereas *Fusobacteriota*, *Actinobacteriota*, *Fusobacterium*, and *Moraxellaceae* were abundant in the NR+DSS+IFX group (*p* < 0.05; Figure 2D). Compared with the R+DSS+IFX group, the abundances of *Acidobacteriota*, *Proteobacteria*, *Actinobacteriota*, and *Fusobacteriota* were significantly higher, and the abundances of *Firmicutes* and *Bacteroidota* were significantly lower in the NR+DSS+IFX group (*p* < 0.05; Figure 2E). At the genus level, the abundances of *Nitrosomonadaceae*, *Lactobacillus*, *Fusobacterium*, and *Bamesiella* significantly increased in the NR+DSS+IFX group, whereas the abundances of *Blautia*, *Oscillibacter*, and *Papillibacter* significantly decreased compared with those in the R+DSS+IFX group (*p* < 0.05; Figure 2F–K). These data indicate that FMT alters the gut microbiota diversity in mice with DSS‐induced colitis.

### 2.3 The Gut Microbiota in the Tissues of Patients with UC is Associated with IFX Response

**Figure 3:**
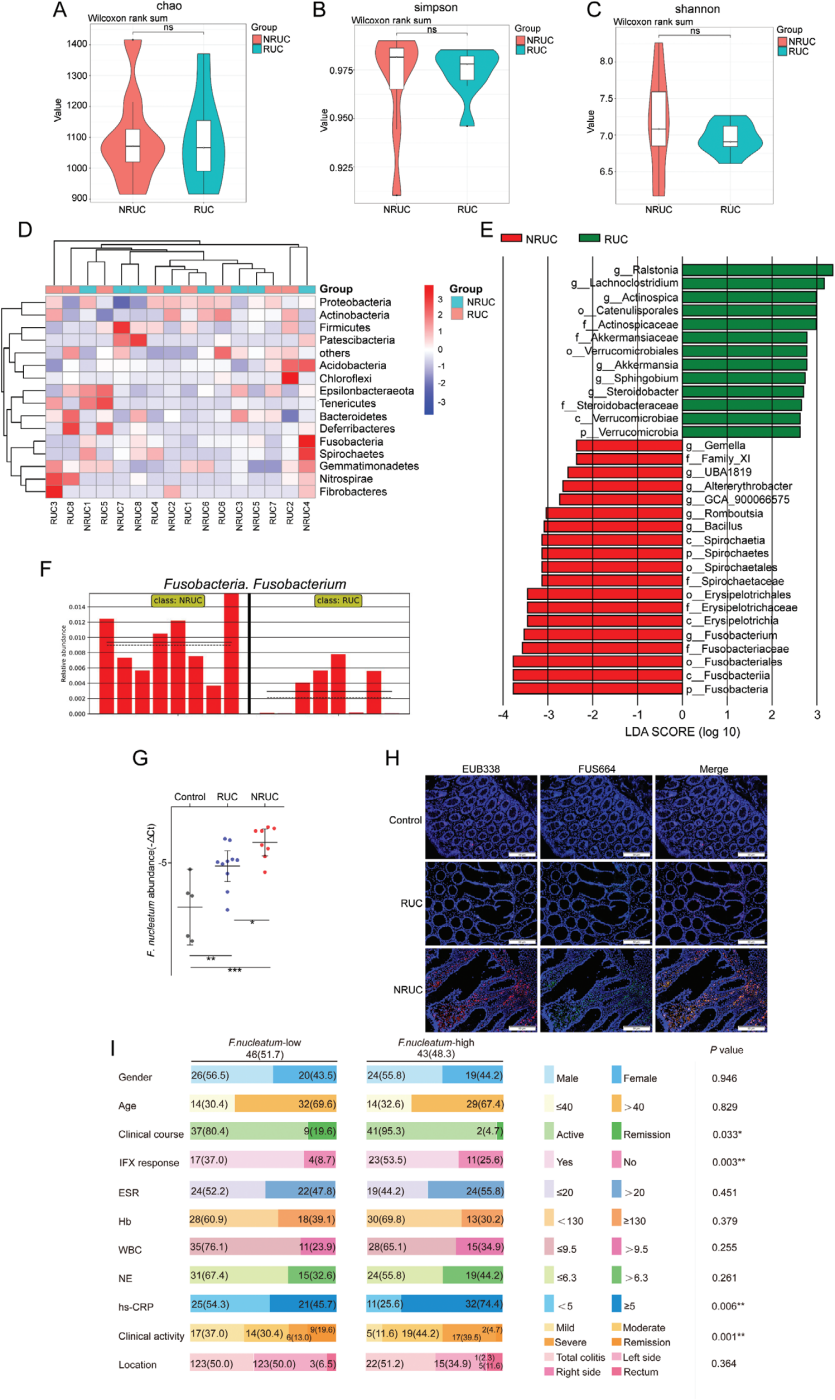
Changes of microbial diversity in intestinal tissues of UC patients. A–C) Alpha diversity boxplot. D) Hierarchically clustered heat map analysis of the highly represented bacterial taxa (phylum level) in tissues from RUC and NRUC patients by 16S rDNA sequencing. E) LEfSe analysis identifies the relative taxa abundance from RUC (green) and NRUC (red) patients. F) The abundance of *Fusobacterium* from RUC and NRUC tissues. G) Statistical analysis of the abundance of *F. nucleatum* in RUC (*n* = 10) and NRUC (*n* = 8) patients (*p < 0.05, and **p < 0.01, and ***p < 0.001; the dividing lines indicate any joins; nonparametric Mann‐Whitney U test. Error bars indicate SD). H) Representative images of FISH to assess the amount of *F. nucleatum* in RUC tissues (*n* = 40), NRUC tissues (*n* = 15), and control. EUB338 (red) is a Cy3‐conjugated “universal bacterial” oligonucleotide probe; FUS664 (green) is a FITC‐conjugated *F. nucleatum* oligonucleotide probe. 200 × magnification. I) Stratification of *F. nucleatum* abundance in 89 UC patients and its significant association with clinicopathological factors.

Considering the differences in the microbiota between the patients’ feces and tissues, as well as the abundance of bacteria colonized from the patients’ stool in the gut of the mice, we further analyzed the bacterial composition in the intestinal tissues of NRUC (n = 8) and RUC patients (n = 8) via 16S rDNA sequencing. There was no statistically significant difference in the alpha diversity of the gut microbiota in the intestinal tissues of these patients (*p* > 0.05; **Figure** **3**A–C). Compared with those in RUC patients, the abundances of *Fusobacteriota*, *Patescibacteria*, and *Spirochaetes* were significantly higher in the tissues of NRUC patients, whereas the abundances of *Chloroflexi*, *Deferribacteres*, and *Tenericutes* were significantly lower (*p* < 0.05; Figure 3D). Further linear discriminant analysis revealed that the abundances of *Romboutsia* and *Fusobacterium* in NRUC patients were significantly higher than in RUC patients. In contrast, the abundances of *Akkermansia*, *Ralstonia*, *Lachnoclostridium*, and *Steroidobacter* decreased significantly (*p* < 0.05; Figure 3E,F)

Given the increased abundance of *Fusobacterium* in the intestinal tissues of NR+DSS+IFX mice and NRUC patients (Figure S2, Supporting Information) and the role of *Fusobacterium*, especially *F. nucleatum*, in UC progression, we further explored the abundance of *F. nucleatum* in UC tissues. Consistent with the sequencing data, RT‐PCR data revealed that *F. nucleatum* was more highly enriched in UC tissues than in normal tissues (n = 5; *p* < 0.01; Figure 3G). Furthermore, the abundance of *F. nucleatum* in NRUC tissues (n = 8) was higher than that in RUC tissues (n = 10) (*p* < 0.05; Figure 3G). We further detected *F. nucleatum* abundance in the tissues of 89 patients with UC, including 40 RUC patients and 15 NRUC patients, using fluorescence in situ hybridization (FISH) to investigate the role of *F. nucleatum* in anti‐TNF therapy in patients with UC. *F. nucleatum* was detected in a higher percentage of NRUC tissues (73.33%) than in RUC tissues (42.5%; *p* < 0.05; Figure 3H). Next, we evaluated the relationship between *F. nucleatum* abundance and the clinicopathological features of patients. The abundance of *F. nucleatum* was associated with the clinical course, IFX response, clinical activity, and hs‐CRP levels (*p* < 0.05; Figure 3I). These data suggest that *F. nucleatum* abundance is possibly associated with the therapeutic responsiveness of UC to IFX.

### 2.4 *F. nucleatum* Inhibits the Therapeutic Response of DSS‐induced Colitis Mice to IFX

**Figure 4:**
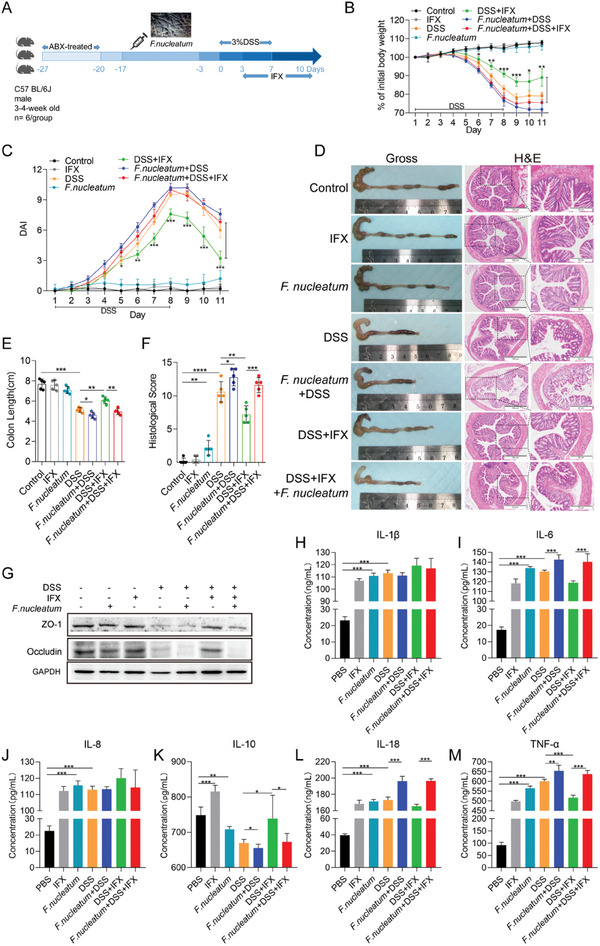
*F. nucleatum* inhibits the therapeutic response of DSS‐induced colitis to IFX. A) Schematic representation of experimental setup for mice. B,C) Statistical analysis of body weight change B) and the disease activity index (DAI) C) of mice (*n* = 5 per group; *p < 0.05, **p < 0.01, and ***p < 0.001; the dividing lines indicate any joins; one‐way ANOVA combined with Bonferroni's *post hoc* test. Error bars indicate SD). D) Representative image of colons and H&E staining of colon sections from mice of each group (4 × magnification and 200 × magnification). E,F) Statistical analysis of colon length E) and histopathological score of the colon F) (*n* = 5 per group; *p < 0.05, **p < 0.01, and ***p < 0.001; the dividing lines indicate any joins; nonparametric Mann‐Whitney U test. Error bars indicate SD). G) Western blotting was performed to detect ZO‐1 and Occludin expression in tissues of different groups of mice. H–M) ELISA detected the expression of inflammatory factors in the serum of different groups of mice (*n* = 5 per group; *p < 0.05, **p < 0.01, and ***p < 0.001; the dividing lines indicate any joins; nonparametric Mann‐Whitney U test. Error bars indicate SD).

To investigate whether *F. nucleatum* affects the therapeutic responsiveness to IFX in vivo, we administered *F. nucleatum* by gavage to ABX‐treated mice, examined the colonization of *F. nucleatum* (Figure S3, Supporting Information), and treated them with DSS or IFX (**Figure** **4**A). We found that the DSS and IFX groups presented slower body weight loss (*p* < 0.01; Figure 4B), lower DAI (*p* < 0.01; Figure 4C), lower colon size ratio (*p* < 0.05; Figure 4E), and lower HS (*p* < 0.05; Figure 4F) compared with the DSS group. After *F. nucleatum* infection, weight loss, cecal edema, colon shortening, and colitis significantly increased in the DSS+IFX group (*p* < 0.01; Figure 4B–F). Additionally, western blotting revealed that the expression levels of ZO‐1 and Occludin in the intestinal tissues of the mice in the DSS+IFX group were significantly higher than those in the DSS group (*p* < 0.05; Figure 4G). However, the expression levels of ZO‐1 and Occludin in the intestinal tissues of the *F. nucleatum*+DSS+IFX group were significantly lower than those in the intestinal tissues of the DSS+IFX group (*p* < 0.01; Figure 4G). These data indicate that *F. nucleatum* may affect the therapeutic response to IFX in DSS‐induced colitis mice.

Moreover, we detected the secretion of inflammatory cytokines in the serum of the mice in each group (Figure 4H–M). Compared with that in the DSS group, the secretion of TNF‐α in the serum of the DSS+IFX group was significantly reduced (*p* < 0.001; Figure 4M), whereas the secretion of IL‐10 was increased (*p* < 0.05; Figure 4K), indicating that IFX could inhibit the secretion of TNF‐α in DSS‐induced colitis. However, compared with those in the DSS+IFX group, the secretion of IL‐6, IL‐18, and TNF‐α in the serum of the *F. nucleatum*+DSS+IFX group was significantly higher (*p* < 0.001; Figure 4I,L,M), while the secretion of IL‐10 was lower (*p* < 0.05; Figure 4K). These results suggest that *F. nucleatum* increases the secretion of relevant inflammatory cytokines, thereby affecting the responsiveness of DSS‐induced colitis to IFX treatment.

### 2.5 *F. nucleatum* Promotes Resistance to Anti‐TNF Therapy in DSS‐Induced Colitis through Activating the p38 MAPK Signaling Pathway

Recent studies have suggested that immune cell infiltration plays a significant role in the development of UC.^[^ ^22^ ^]^ Macrophages, activated dendritic cells, and neutrophils play crucial roles in the occurrence and progression of UC.^[^ ^23^ ^]^ To verify the correlation between immune cell infiltration and the abundance of *F. nucleatum* in UC patient tissues, we used immunofluorescence and found that a high abundance of *F. nucleatum* was accompanied by a significant increase in CD68‐positive macrophage infiltration (*p* < 0.01) and a significant decrease in CD83‐positive dendritic cells (*p* < 0.05; Figure S4A,B, Supporting Information) in UC tissues. These data suggest that *F. nucleatum* abundance is associated with macrophage infiltration in the tissues of patients with UC. RNA‐seq was then performed to analyze the gene expression profiles of THP‐1 cells co‐cultured with or without *F. nucleatum*. In total, 1939 upregulated genes and 1155 downregulated genes were detected (adjusted *p* < 0.05). Single‐sample gene set enrichment analysis (ssGSEA) revealed that differential gene enrichment was not significant in *F. nucleatum*‐infected THP‐1 cells (Figure S4C, Supporting Information). These data indicate that *F. nucleatum* affects UC disease progression independent of immune cells.

**Figure 5:**
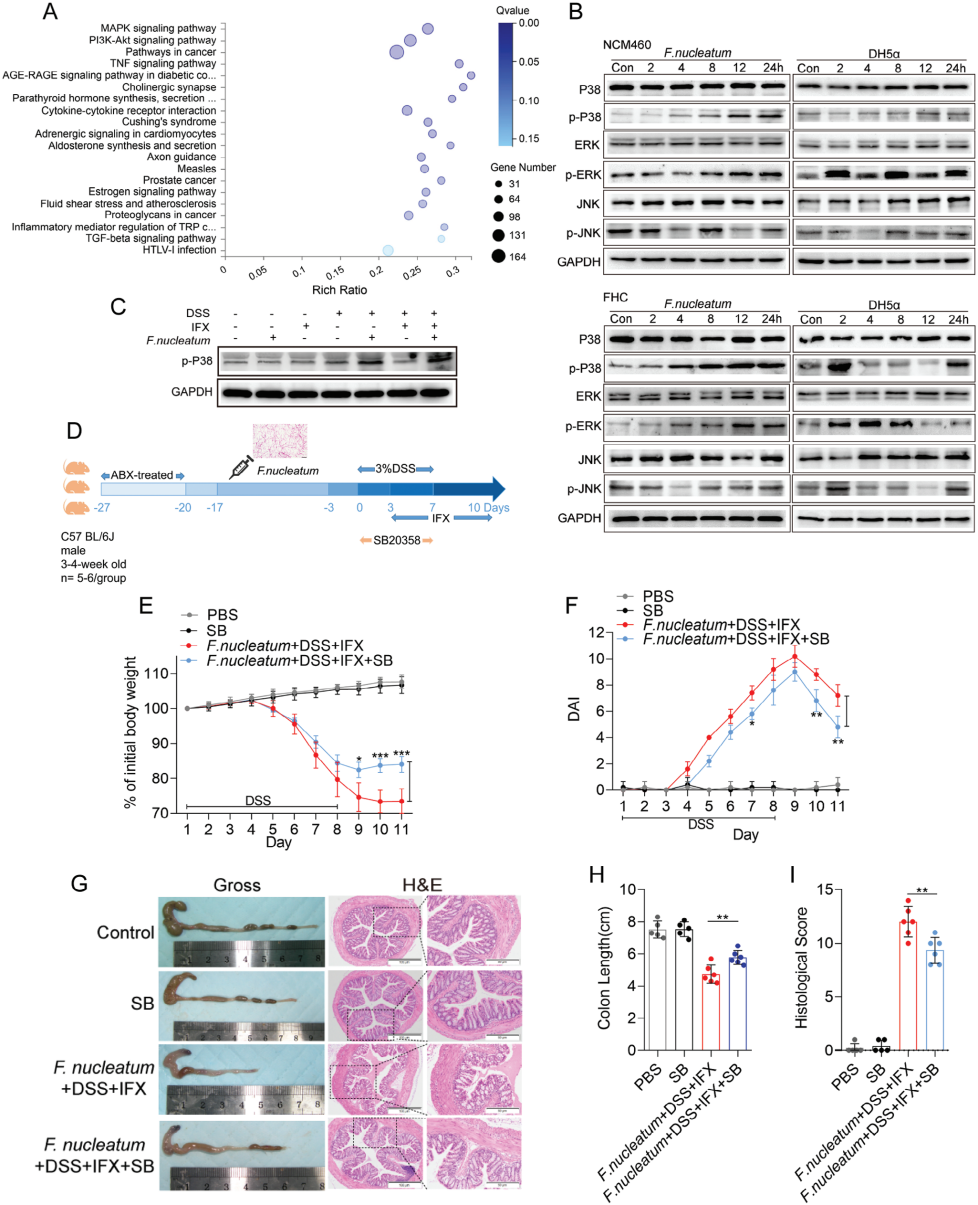
*F.nucleatum* affects the therapeutic response of DSS‐induced colitis to IFX through the P38 MAPK signaling pathway. A) ssGSEA was conducted to reveal the relationship between the abundance of *F. nucleatum* and the related pathway activity in NCM460 cells. B) Western blot analysis was performed to detect MAPK element expression in NCM460 and FHC cells co‐cultured with *F. nucleatum*, *E. coli*, or PBS (control). C) Western blot analysis was performed to detect p‐p38 expression in mice treated with *F. nucleatum*, DSS, or IFX. D) Schematic representation of experimental setup for mice. E,F) Statistical analysis of body weight change E) and DAI F) of mice (*n* = 5–6 per group; *p < 0.05, **p < 0.01, and ***p < 0.001; the dividing lines indicate any joins; one‐way ANOVA combined with Bonferroni's *post hoc* test. Error bars indicate SD). G) Representative image of colons and H&E staining of colon sections from mice of each group (4 × magnification and 200 × magnification). H,I) Statistical analysis of colon length H) and histopathological score of the colon I) (*n* = 5–6 per group; *p < 0.05, **p < 0.01, and ***p < 0.001; the dividing lines indicate any joins; nonparametric Mann‐Whitney U test. Error bars indicate SD).

Furthermore, we investigated the effects of *F. nucleatum* infection on intestinal epithelial cells. Transcriptome sequencing revealed that *F. nucleatum* significantly upregulated the expression of 2596 genes and downregulated the expression of 1253 genes in NCM460 cells (adjusted *p* < 0.05). ssGSEA revealed that after *F. nucleatum* infection, genes associated with the MAPK, PI3K‐AKT, and TNF signaling pathways were significantly enriched in NCM460 cells (adjusted *p* < 0.01; **Figure** **5**A). To test these results, we incubated NCM460 cells and fetal human colon (FHC) cells with *F. nucleatum*, *E. coli* (DH5α), or PBS (control, Con). Compared with *E. coli* and PBS treatment, *F. nucleatum* treatment substantially increased the phosphorylation level of p38 in a time‐dependent manner (*p* < 0.01), whereas there was no significant difference in the phosphorylation levels of JNK and ERK (*p* > 0.05; Figure 5B; *E. coli* or PBS as the control). These data suggest that *F. nucleatum* activates the MAPK signaling pathway in epithelial cells. Moreover, we detected the expression of phosphorylated p38 in *F. nucleatum* or IFX‐treated DSS‐induced colitis mice. Western blotting revealed a lower level of phosphorylated p38 in colitis tissues from DSS+IFX mice than in those from DSS mice. Compared with the DSS+IFX group, the *F. nucleatum*+DSS+IFX group showed significantly increased expression of phosphorylated p38 (*p* < 0.05; Figure 5C). These results suggest that *F. nucleatum* promotes resistance to IFX through activating the p38 MAPK signaling pathway.

To verify whether *F. nucleatum* affects the therapeutic responsiveness to IFX via the p38 MAPK signaling pathway in vivo, ABX‐treated mice were initially administered *F. nucleatum* and then subjected to DSS, IFX, or the p38 signaling pathway activation‐specific inhibitor adezmapimod (SB 203580) (Figure 5D). We found that mice in the *F. nucleatum*+DSS+IFX+SB group presented slower body weight loss (*p* < 0.001; Figure 5E), lower DAI (*p* < 0.01; Figure 5F), and lower colon size ratio (*p* < 0.01; Figure 5H) than mice in the *F. nucleatum*+DSS+IFX group. Further observation of colonic histopathological sections and HE staining revealed that, compared with the *F. nucleatum*+DSS+IFX group, the *F. nucleatum*+DSS+IFX+SB group presented less epithelial damage (Figure 5G), including mucosal erosion, crypt loss, and lymphocyte infiltration, and lower HS (*p* < 0.01; Figure 5I). These results indicate that inhibiting of the p38 MAPK signaling pathway alleviates the resistance of DSS‐induced colitis mice to IFX treatment after *F. nucleatum* intervention.

### 2.6 *F. nucleatum* is Associated with Epithelial NAD^+^ Metabolism

**Figure 6:**
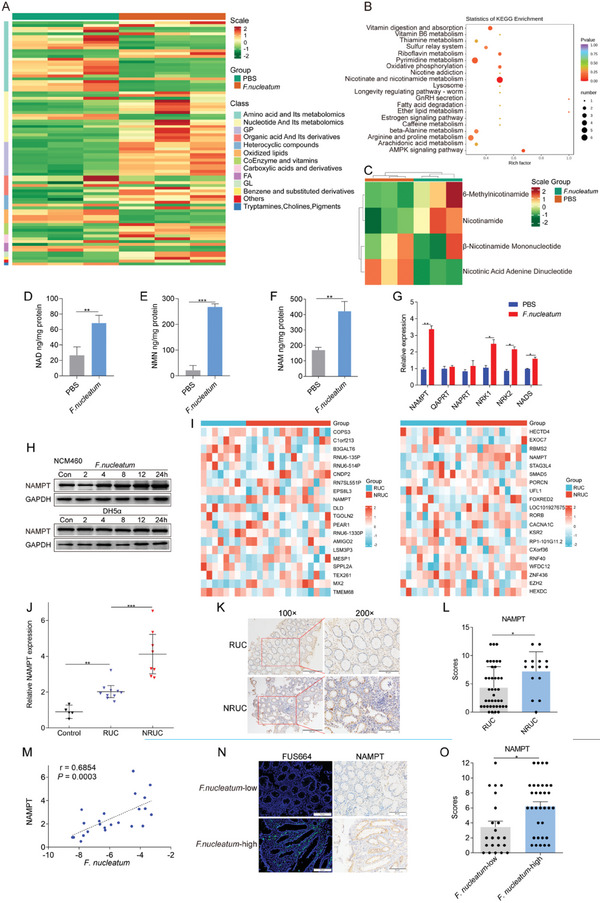
*F. nucleatum* infection is associated with epithelial NAD^+^ metabolism. A) Metabolomics analysis of cells co‐cultured with *F. nucleatum* or PBS (control). B) KEGG enrichment of differential metabolites in NCM460 cells co‐cultured with *F. nucleatum*. C) NAD^+^ related metabolites in NCM460 cells co‐cultured with *F. nucleatum* or PBS (control). D–F) Statistical analysis of NAD^+^, NMN, NAM content in NCM460 cells co‐cultured with *F. nucleatum* or PBS (control) (*p < 0.05, **p < 0.01, and ***p < 0.001; unpaired Student's *t*‐test; Error bars indicate SD). G) NAD^+^ related key enzymesanalysis in NCM460 cells co‐cultured with *F. nucleatum* or PBS (control) (*p < 0.05, **p < 0.01, and ***p < 0.001; unpaired Student's *t*‐test; Error bars indicate SD). H) Western blot analysis was performed with NCM460 cells co‐cultured with *F. nucleatum*, *E. coli*, or PBS (control). I) GSE analysis for NAMPT expression with anti‐TNF resistance in UC using the GSE12251 and GSE73661 datasets. J) Statistical analysis of NAMPT mRNA expression in tissues from UC patients (10 RUC and 8 NRUC) and 5 healthy controls by RT‐PCR (*p < 0.05, **p < 0.01, and ***p < 0.001; the dividing lines indicate any joins; nonparametric Mann‐Whitney U test. Error bars indicate SD). K,L) Representative images of NAMPT expression are shown in K) (100 × magnification and 200 × magnification) and are quantified in L) (*p < 0.05; unpaired Student's *t*‐test; Error bars indicate SD). M) Correlation analysis of NAMPT expression and the abundance of *F. nucleatum* in UC tissues (*r* = 0.6854, *P* = 0.0003; two‐tailed, nonparametric Spearman correlation). N,O) Representative images showing that the abundance of invasive *F. nucleatum* in UC tissues is associated with high expression of NAMPT N) (200 × magnification). The NAMPT protein levels in 22 *F. nucleatum*‐low and 33 *F. nucleatum*‐high UC tissues from patients were measured by immunohistochemical analysis (O; *p < 0.05; unpaired Student's *t*‐test; Error bars indicate SD).

Next, we performed metabolomic analysis to assess whether *F. nucleatum* infection affected the metabolites in intestinal epithelial cells. A total of 53 significantly upregulated metabolites and 34 downregulated metabolites were detected (fold change ≥ 2; Table S1 and Figure S5A, Supporting Information). Among the primary metabolites, the contents of most metabolites such as nucleotide and its metabolomics, glycerol phosphatide (GP), coenzyme and vitamins, glycerides (GL), and benzene and substituted derivatives were significantly increased. In contrast, the levels of most metabolites in tryptamines, cholines, pigments, amino acids, and their metabolomics, as well as fatty acids (FA) decreased (**Figure** **6**A). The Kyoto Encyclopedia of Genes and Genomes (KEGG) pathway enrichment analysis revealed that compared with those in the PBS group, the differentially abundant metabolites in NCM460 cells after *F. nucleatum* infection were mainly enriched in the nicotinate and nicotinamide metabolism pathways (adjusted *p* < 0.05; Figure 6B; Figure S5B, Supporting Information), suggesting that *F. nucleatum* is associated with nicotinate and nicotinamide metabolism in intestinal epithelial cells. We further analyzed the content of nicotinamide‐related metabolites. The results showed that nicotinamide and 6‐methylnicotinamide levels in NCM460 cells treated with *F. nucleatum* were higher than in the PBS group. In contrast, the level of nicotinic acid adenine dinucleotide was lower in the *F. nucleatum*‐treated group than in the PBS group (Figure 6C).

Nicotinamide is the main precursor of NAD^+^. The content of nicotinic acid adenine dinucleotide, a key metabolite of the Preiss‐handler pathway and *de novo* synthesis pathway, was significantly reduced, suggesting that *F. nucleatum* may participate in the NAD^+^ salvage pathway in epithelial cells. High‐performance liquid chromatography (HPLC) was subsequently conducted to assess the levels of niacinamide (NAM), NAD^+^, and β‐nicotinamide mononucleotide (NMN) in intestinal epithelial cells after infection with *F. nucleatum*. Compared with the PBS group, *F. nucleatum* infection increased the levels of NAM, NMN, and NAD^+^ in NCM460 cells (*p* < 0.01; Figure 6D–F). Furthermore, we verified the changes in the expression of key enzymes in the NAD^+^ synthesis pathway via RT‐PCR, which revealed that *F. nucleatum* infection upregulated the expression of the *NAMPT*, *NRK1*, *NRK2*, and *NADS* genes in NCM460 cells (*p* < 0.05; Figure 6G). Consistent with this finding, western blotting revealed that the *F. nucleatum*‐mediated increase in the level of NAMPT was time‐dependent (*p* < 0.05; Figure 6H). Taken together, these results suggest that *F. nucleatum* promotes the NAD^+^ salvage pathway in intestinal epithelial cells.

We performed gene set enrichment analysis (GSEA) on the GSE12251 and GSE73661 datasets to evaluate whether IFX treatment responsiveness was associated with NAMPT expression in patients with UC. We found that *NAMPT* expression in tissues from NRUC patients was increased compared with tissues from RUC patients (*p* < 0.01; Figure 6I). Next, we examined the expression of *NAMPT* in 5 normal tissues, 8 NRUC tissues, and 10 RUC tissues from the same patients referenced in Figure 3G. Real‐time PCR results revealed that *NAMPT* expression in UC tissues was increased compared with normal tissues (*p* < 0.01; Figure 6J) and was higher in NRUC tissues than in RUC tissues (*p* < 0.001; Figure 6J). Immunohistochemistry was used to further validate the correlation between NAMPT expression and anti‐TNF therapeutic responsiveness in UC tissues. Consistent with the above experimental results, immunohistochemistry revealed that NAMPT expression was significantly increased in the tissues of NRUC patients (*p* < 0.05; Figure 6K,L). Next, to determine whether *F. nucleatum* affected NAMPT expression, we first conducted a correlation analysis. We found that the abundance of *F. nucleatum* (Figure 3G) was positively correlated with *NAMPT* mRNA expression (r = 0.6854, *p* < 0.0003, Figure 6M). We then used FISH and IHC to validate the correlation between the abundance of *F. nucleatum* and NAMPT expression in UC tissues. A high abundance of *F. nucleatum* was often accompanied by high levels of NAMPT (*p* < 0.05; Figure 6N,O). These data suggest that *F. nucleatum* affects anti‐TNF‐α therapeutic responsiveness through NAMPT, a key enzyme of the NAD^+^ salvage pathway.

### 2.7 *F. nucleatum* Activates the p38 MAPK Signaling Pathway by Regulating the NAD^+^ Salvage Pathway to Inhibit Anti‐TNF Drug Therapy Responsiveness

**Figure 7:**
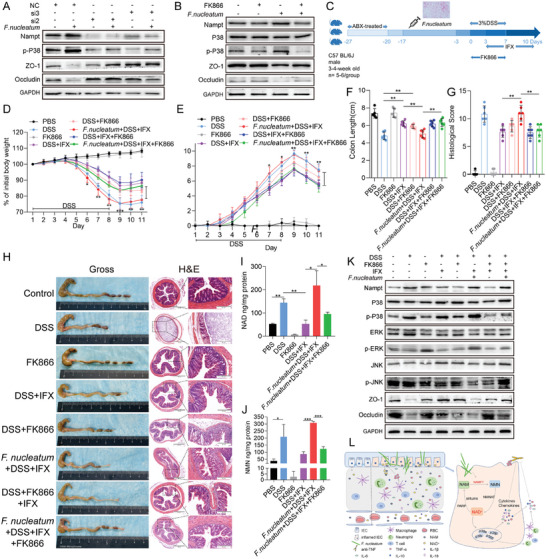
*F.nucleatum* affects the therapeutic response to IFX through NAD^+^ salvage synthesis pathway. A) Western blot analysis was performed with NCM460 cells transfected with siNAMPT and co‐cultured with *F. nucleatum* or PBS (control). B) Western blot analysis was performed with NCM460 cells treated with FK866 and co‐cultured with *F. nucleatum* or PBS (control). C) Schematic representation of experimental setup for mice. D,E) Statistical analysis of body weight change D) and DAI E) of mice (*n* = 5–6 per group; *p < 0.05, **p < 0.01, and ***p < 0.001; the dividing lines indicate any joins; one‐way ANOVA combined with Bonferroni's *post hoc* test. Error bars indicate SD). F,G) Statistical analysis of colon length F) and histopathological score of the colon G) (*n* = 5–6 per group; *p < 0.05, **p < 0.01, and ***p < 0.001; the dividing lines indicate any joins; nonparametric Mann‐Whitney U test. Error bars indicate SD). H) Representative image of colons and H&E staining of colon sections from mice of each group (4 × magnification and 200 × magnification). I,J) Statistical analysis of the content of NAD and NMN in intestinal epithelial cells isolated from mice (*n* = 5–6 per group; *p < 0.05, **p < 0.01, and ***p < 0.001; the dividing lines indicate any joins; nonparametric Mann‐Whitney U test; Error bars indicate SD). K) Western blotting was performed to measure the expression of MAPK associated proteins and mucosal barrier associated proteins in colon tissues from mice. L) Proposed mechanism of *F. nucleatum* affecting the therapeutic response of UC to IFX.

To determine whether the NAD^+^ salvage pathway regulates *F. nucleatum*‐mediated p38 MAPK signaling pathway activation, we performed NAMPT‐targeting siRNA transfection or FK866 treatment in NCM460 cells co‐cultured with *F. nucleatum*. Western blotting revealed that *F. nucleatum* induced the upregulation of NAMPT and phosphorylated p38 and the downregulation of ZO‐1 and Occludin, which were blocked in NCM460 cells, upon transfection with NAMPT‐targeting siRNA or pretreatment with FK866 (*p* < 0.05; **Figure** **7**A,B). These results demonstrate that blocking the NAD^+^ salvage pathway reduces the increase in phosphorylated p38 levels and the destruction of the mucosal barrier induced by *F. nucleatum*.

To investigate whether *F. nucleatum* affects the therapeutic responsiveness of colitis to IFX by regulating the NAD^+^ salvage pathway in vivo, we established a DSS‐induced colitis mouse model (Figure 7C). Compared with those in the *F. nucleatum*+DSS+IFX group, mice in the *F. nucleatum*+DSS+IFX+FK866 group exhibited slower body weight loss (*p* < 0.01; Figure 7D), lower DAI (*p* < 0.01; Figure 7E), and lower colon size ratio (*p* < 0.01; Figure 7F). Further observation of colonic histopathological sections and HE staining revealed that compared with the *F. nucleatum*+DSS+IFX group, the *F. nucleatum*+DSS+IFX+FK866 group presented a lower HS (*p* < 0.01; Figure 7G) and less epithelial damage (Figure 7H), including mucosal erosion, crypt loss, and lymphocyte infiltration. Western blotting revealed lower levels of phosphorylated p38 and phosphorylated ERK and higher levels of ZO‐1 and Occludin in colitis tissues from *F. nucleatum*+DSS+IFX+FK866 mice than in those from *F. nucleatum*+DSS+IFX mice (*p* < 0.05; Figure 7K). These data indicate that inhibiting the NAD^+^ salvage pathway in vivo could alleviate the resistance of mice with DSS‐induced colitis to IFX treatment after *F. nucleatum* infection.

To test the levels of metabolites associated with the NAD^+^ salvage pathway, in situ, mouse intestinal epithelial cells were isolated and analyzed using HPLC. Compared with those in the PBS group, the levels of NAD^+^ and precursor NMN in primary intestinal epithelial cells in the DSS group were significantly higher (*p* < 0.05; Figure 7I,J). The NAD^+^ content in the intestinal primary epithelial cells of mice with DSS‐induced colitis decreased after IFX treatment (*p* < 0.01; Figure 7I). Moreover, NAD^+^ and precursor NMN levels in the epithelial cells of the *F. nucleatum*+DSS+IFX+FK866 group were significantly lower than those in the *F. nucleatum*+DSS+IFX group (*p* < 0.05; Figure 7I,J). These results demonstrate that *F. nucleatum* regulates the levels of metabolites associated with the NAD^+^ salvage pathway in vivo.

Furthermore, we measured the levels of inflammatory factors in the serum of the model mice using ELISA. Compared with those in control mice, several inflammatory factors in the serum of DSS‐treated mice were upregulated, including IL‐1β, IL‐6, IL‐8, IL‐18, and TNF‐α, or downregulated, including IL10 (*p* < 0.001; Figure S6A–F, Supporting Information). After treatment with IFX, the secretion of IL‐1β, IL‐6, and TNF‐α decreased, whereas the secretion of IL‐10 increased in the serum of DSS‐treated mice (*p* < 0.001; Figure S6A,B,D,F, Supporting Information). In addition, compared with those in the *F. nucleatum*+DSS+IFX group, the levels of IL‐1β, IL‐6, IL‐8, IL‐18, and TNF‐α in the serum of the *F. nucleatum*+DSS+IFX+FK866 group were lower (*p* < 0.01; Figure S6A–F, Supporting Information). RT‐PCR was performed to measure the levels of inflammatory factor mRNAs in the intestinal tissues of the mice. The results revealed that the expression of IL‐1β, IL‐6, and TNF‐α in the DSS group significantly increased, whereas the expression of IL‐10 was decreased compared with that in the control group (*p* < 0.05; Figure S6G–J, Supporting Information). The expression of IL‐1β and TNF‐α was reduced in the intestinal tissues of DSS‐treated mice after IFX treatment (*p* < 0.05; Figure S6G,J, Supporting Information). Moreover, the expression of IL‐6 and TNF‐α was increased in the *F. nucleatum*+DSS+IFX group compared with the DSS+IFX group. In contrast, TNF‐α expression was lower in the *F. nucleatum*+DSS+IFX+FK866 group than in the *F. nucleatum*+DSS+IFX group (*p* < 0.05; Figure S6J, Supporting Information). These data suggest that *F. nucleatum* influences the responsiveness to anti‐TNF therapy through secreting inflammatory factors.

In summary, we conclude that *F. nucleatum* promotes the NAD^+^ salvage pathway by upregulating NAMPT expression, which subsequently leads to the activation of the p38 MAPK signaling pathway and then promotes the secretion of inflammatory factors, ultimately inhibiting the therapeutic response to anti‐TNF drugs.

## 3 Discussion

Anti‐TNF therapies are recommended for patients with moderate‐to‐severe UC who do not respond to conventional therapies or cannot receive such therapies because of unacceptable side effects. However, 50% of patients with UC do not respond to anti‐TNF therapy or lose their responsiveness over time.^[^ ^24^ ^]^ At present, there are many biomarkers for evaluating the response of IBD to anti‐TNF therapy;^[^ ^25^, ^26^ ^]^ however, most of these biomarkers lack robust validation.^[^ ^27^ ^]^ Recent studies suggest that the gut microbiota plays an important role in the response to anti‐TNF therapy,^[^ ^3^, ^4^ ^]^ highlighting the potential of microbiome monitoring as a companion diagnostic approach.^[^ ^28^ ^]^ Therefore, exploring the mechanisms underlying microbiological factor‐associated resistance in IBD is particularly important for the development of current therapeutic strategies.

FMT overcomes anti‐PD‐1 therapy resistance in melanoma patients.^[^ ^29^ ^]^ However, whether FMT can be used as an alternative conversion therapy for patients who are unresponsive to IFX remains unknown. One study has reported that immune‐mediated colitis patients who failed to respond to immunosuppression (infliximab or vedolizumab) received FMT from healthy donors as salvage therapy. Most patients achieved clinical remission at the end of the study period.^[^ ^30^ ^]^ Moreover, UC patients who lose responsiveness to anti‐TNF therapy show improvements in symptoms and mucosal inflammation after FMT.^[^ ^31^ ^]^ In another study of patients with prior failure of infliximab in CD who received FMT as a switch therapy, those who received FMT had significantly lower rates of clinical relapse compared with patients who received IFX treatment.^[^ ^32^ ^]^ These studies indicate that FMT affects the efficacy of anti‐TNF therapy in IBD patients. Consistent with previous studies, our study also suggests that the gut microbiota can affect the response to anti‐TNF therapy in colitis. Herein, we performed FMT in ABX model mice using feces from RUC patients and NRUC patients and found that, compared with the R+DSS+IFX group, the NR+DSS+IFX group exhibited more severe disease and disruption of mucosal structures. These findings indicate that transplantation of gut fecal microbiota from UC patients of different origins leads to different responses to IFX treatment in mice with DSS‐induced colitis. Compared with the DSS+IFX group, the NR+DSS+IFX group showed rapid weight loss, higher DAI, shorter colon length, and higher HS, suggesting that transplanting the gut microbiota of NRUC patients may reduce the efficacy of IFX therapy in DSS‐induced colitis mice. In contrast to previous studies, we used feces from UC patients to perform FMT in ABX model mice, which could better mimic the intestinal microbial environment of patients. In addition, we simultaneously transplanted fecal microbiota from RUC and NRUC patients. The results showed that fecal bacteria from RUC patients did not affect the responsiveness to IFX treatment in mice with DSS‐induced colitis transplanted with fecal bacteria from NRUC patients, possibly because human fecal bacteria are partially located in the gut of mice and because of the difference between the intestinal mucosal flora and fecal bacteria.

Fecal and mucosal microbiota, as well as their endogenous metabolites, could provide a predictive tool for assessing the response of patients with IBD to various biologics.^[^ ^28^, ^33^, ^34^ ^]^ A previous study revealed no difference in the gut bacterial alpha diversity between the stools of CD patients who were responsive to IFX and those who were not responsive to IFX. The relative abundances of several bacterial genera, including *Clostridium XI*, *Clostridium XVIII*, *Eggerthella*, *Lachnospiracea incertae sedis*, *Parabacteroides*, and *Peptococcus*, in IFX non‐responders were significantly higher than those in IFX responders.^[^ ^35^ ^]^ Another study revealed lower dysbiosis indices and a higher abundance of *Faecalibacterium prausnitzii* in UC anti‐TNF therapy responders than nonresponders at baseline.^[^ ^36^ ^]^ In this study, we performed 16S rDNA sequencing to analyze the differences in mucosal microbiota between RUC patients and NRUC patients and found no statistically significant difference in alpha diversity. In contrast to previous studies, our study revealed that the abundance of *Romboutsia* and *Fusobacterium* was significantly higher in the tissues of NRUC patients than in those of RUC patients, whereas the abundance of *Akkermansia*, *Ralstonia*, *Lachnoclostridium*, and *Steroidobacter* was significantly lower, possibly because previous studies analyzed CD samples or fecal samples from patients with UC and did not analyze the microbiota composition in UC intestinal mucosal tissue. In patients with CD, the abundance of *Fusobacteria* was higher in the mucosa of patients in the non‐responsive group than that in the responsive group,^[^ ^7^ ^]^ suggesting that *Fusobacteria* may be associated with anti‐TNF responsiveness in patients with CD. Studies have shown that *F. nucleatum* is highly abundant in the tissues and feces of patients with UC and plays an important role in the development of UC.^[^ ^8^, ^9^, ^10^ ^]^ However, the effect of *F. nucleatum* on the response to anti‐TNF treatment in UC patients has not been reported. In the present study, we used RT‐PCR and FISH to verify the abundance of *F. nucleatum* in UC tissues and reported a significant increase in the abundance of *F. nucleatum* in NRUC tissues compared with that in RUC tissues. Furthermore, we generated DSS‐induced colitis model mice and found that *F. nucleatum* exacerbated colitis symptoms, including weight loss, cecal edema, colon shortening, and colitis, in IFX‐treated mice. Additionally, the expression levels of ZO‐1 and Occludin in the intestinal tissue of the *F. nucleatum*+DSS+IFX group were significantly lower than those in the intestinal tissue of the DSS+IFX group. Therefore, our study revealed that the abundance of *F. nucleatum* in tissues might be correlated with the response of patients with UC to anti‐TNF therapy and that *F. nucleatum* could inhibit the responsiveness of DSS‐induced colitis mice to anti‐TNF therapy.

NAD^+^ is an important coenzyme in biometabolic processes and is synthesized via three distinct pathways: the NAD^+^ salvage, Preiss‐Handler, and *de novo* pathways, with the salvage pathway being the predominant source of NAD^+^.^[^ ^14^ ^]^ In salvage pathway, nicotinamide is converted to NMN through the enzymatic reaction of NAMPT, the rate‐limiting enzyme of the NAD^+^ salvage pathway. In several disorders, including arthritis and psoriasis, the NAMPT‐mediated NAD^+^ salvage pathway is thought to have inflammatory function,^[^ ^37^, ^38^ ^]^ indicating that the NAD^+^ salvage pathway is associated with inflammation. NAMPT is also associated with intestinal inflammation in patients with IBD^[^ ^14^ ^]^ and is correlated with the response to anti‐TNF therapy in these patients.^[^ ^16^, ^17^ ^]^ In this study, we verified the changes in the expression of key enzymes of the NAD^+^ synthesis pathway via RT‐PCR, which revealed that *F. nucleatum* infection upregulated the expression of the *NAMPT*, *NRK1*, *NRK2*, and *NADS* genes. In contrast, the expression of NAPRT and QAPRT, which are key enzymes of the Preiss‐Handler and *de novo* pathways, respectively, did not increase. Moreover, we found that the expression of NAMPT was significantly higher in the tissues of NRUC patients than in those of RUC patients and that *F. nucleatum* abundance was positively associated with NAMPT expression in UC tissues. NAD^+^ regulates glycolysis, the tricarboxylic acid cycle, and oxidative phosphorylation‐driven energy metabolism through redox reactions.^[^ ^39^ ^]^ Recent studies have shown that *F. nucleatum* can increase intracellular glycolysis in colon cancer cells to promote tumorigenesis.^[^ ^40^ ^]^ In addition, *F. nucleatum* is positively correlated with the levels of several plasma lipid metabolites^[^ ^41^ ^]^ and plays a key role in lipid homeostasis in the apoptosis induced by NAMPT inhibitors,^[^ ^42^ ^]^ suggesting that *F. nucleatum* may be involved in glycolipid metabolism. In this study, we performed metabolomic analysis to detect the metabolites of epithelial cells infected with *F. nucleatum*. We found that the contents of most metabolites in the nucleotides and their metabolites, glycerol phosphatides, coenzymes and vitamins, glycerides, benzene, and substituted derivatives were significantly increased. Furthermore, differentially abundant metabolites were mainly enriched in nicotinate and nicotinamide metabolism in epithelial cells infected with *F. nucleatum*. Additionally, the levels of NAM, NMN, and NAD^+^ increased, and the expression of the key enzyme NAMPT in the NAD^+^ salvage pathway increased in intestinal epithelial cells after infection with *F. nucleatum*. Thus, our study suggests that *F. nucleatum*, which is associated with anti‐TNF resistance, promots the NAD^+^ salvage pathway in intestinal epithelial cells by upregulating the key enzyme NAMPT. However, we did not elucidate the mechanism by which *F. nucleatum* regulates NAMPT expression. *F. nucleatum* inhibits butyric acid to promote tumor progression via the AMPK signaling pathway.^[^ ^43^ ^]^ Previous studies have demonstrated that intestinal AMPK is a possible upstream mediator of intestinal NAMPT‐mediated NAD^+^ biosynthesis and may play a central role in maintaining intestinal homeostasis, including microbial composition.^[^ ^44^ ^]^ Therefore, *F. nucleatum* might affect NAMPT‐mediated NAD^+^ biosynthesis by regulating AMPK. However, further studies are needed to precisely understand how *F. nucleatum* regulates the expression of NAMPT.

Previous studies have shown that *F. nucleatum* can invade cells to activate the p38 MAPK signaling pathway and promote the secretion of inflammatory factors, such as IL‐8.^[^ ^45^, ^46^ ^]^ The activation of the p38 MAPK pathway is associated with the efficacy of anti‐TNF therapy.^[^ ^18^, ^19^ ^]^ Consistent with the findings of previous studies, in this study, we performed transcriptome sequencing and found that genes that were differentially expressed in intestinal epithelial cells infected with *F. nucleatum* were significantly enriched in the MAPK signaling pathway. NCM460 cells and FHC cells were incubated with *F. nucleatum*, *E. coli* or PBS. Compared with *E. coli* and PBS treatment, the phosphorylation level of p38 was increased in a time‐dependent manner after *F. nucleatum* infection, suggesting that *F. nucleatum* can activate the p38 MAPK signaling pathway in vitro. Furthermore, we treated ABX mice with DSS, IFX, SB, and *F. nucleatum*. We found that compared with *F. nucleatum*+DSS+IFX‐treated mice, *F. nucleatum*+DSS+IFX+SB‐treated mice exhibited improved epithelial damage and fewer symptoms of colitis, indicating that inhibition of the p38 MAPK signaling pathway alleviated resistance to IFX treatment in *F. nucleatum*‐infected DSS‐induced colitis mice. Studies have shown that NAMPT promotes p38 MAPK phosphorylation in endothelial cells and that p38 MAPK activation can be suppressed by knocking down NAMPT in senescent cells.^[^ ^20^, ^47^ ^]^ In this study, we found that the increase in p38 phosphorylation and the disruption of the mucosal barrier proteins ZO‐1 and Occludin induced by *F. nucleatum* were blocked by the knockdown or inhibition of NAMPT expression in intestinal epithelial cells. After the NAD^+^ salvage pathway was blocked by FK866 in vivo, mice in the *F. nucleatum*+DSS+IFX group exhibited slower body weight loss, lower DAI, lower colon size ratio, and lower HS than those in the *F. nucleatum*+DSS+IFX group. Moreover, the levels of phosphorylated p38 and phosphorylated ERK decreased, and the expression of ZO‐1 and Occludin increased in colitis tissues in the *F. nucleatum*+DSS+IFX+FK866 group. These results suggest that blocking the NAD^+^ salvage pathway could inhibit the activation of the p38 MAPK signaling pathway induced by *F. nucleatum*, thus alleviating the resistance of DSS‐induced colitis mice to IFX treatment. However, further studies are needed to determine whether NAD^+^ metabolism directly regulates the p38 MAPK signaling pathway.

Finally, we explored the effect of *F. nucleatum* on inflammatory factor expression and the correlation between inflammatory factor expression and the response to IFX treatment in UC. Some studies have shown that the expression of inflammatory factors such as IL‐6, IL‐10, IL‐1B, CXCL8, and CCL2 is correlated with the response to anti‐TNF therapy in IBD.^[^ ^48^, ^49^, ^50^ ^]^ Consistent with previous studies, our research revealed that *F. nucleatum* promoted the secretion of IL‐6, IL‐18, and TNF‐α and inhibited the secretion of IL‐10 in the serum of DSS‐induced colitis mice treated with IFX while increasing the expression of IL‐6 and TNF‐α in the colon tissues. Blocking the NAD^+^ salvage pathway inhibited the secretion of IL‐1β, IL‐6, IL‐8, IL‐18, and TNF‐α in the serum and reduced the expression of TNF‐α in the tissues of *F. nucleatum*‐infected IFX‐treated DSS‐induced colitis mice. Further exploration of how these inflammatory factors regulate the response to anti‐TNF therapy and how *F. nucleatum* affects the secretion of these inflammatory factors during drug resistance will be highly important.

The association between *F. nucleatum* and the development of UC has been extensively confirmed, and the potential mechanisms involved have been elucidated. Targeted therapy for *F. nucleatum*, for example by reducing its growth or eliminating it from the gut microbiota without harming beneficial bacteria, is a promising research approach. Probiotics show great potential in the prevention and treatment of many diseases.^[^ ^51^ ^]^ Numerous studies have demonstrated that probiotics, such as *Bifidobacterium*, *Streptococcus thermophilus*,^[^ ^52^ ^]^ *Lactobacillus salivarius*,^[^ ^53^ ^]^ and *Lactobacillus reuter* ^[^ ^54^ ^]^ inhibit *F. nucleatum* in vitro and in vivo. Short‐chain fatty acid butyrate can reduce the colonization and invasion of *F. nucleatum* in colorectal cancer tissues and relieve *F. nucleatum*‐induced chemoresistance.^[^ ^55^ ^]^ In our study, we found that *F. nucleatum* was more abundant in NRUC tissues than in RUC tissues, and the abundance of *F. nucleatum* was associated with IFX responsiveness. After *F. nucleatum* infection, weight loss, cecal edema, colon shortening, and colitis significantly increased in the DSS+IFX group. However, whether these probiotics can alleviate the resistance of UC patients to IFX treatment by inhibiting the growth and colonization of *F. nucleatum* should be verified.

In this study, we revealed that FK866 inhibited the NAD^+^ salvage pathway and alleviated the resistance of mice with DSS‐induced colitis to IFX treatment after *F. nucleatum* infection. A previous study has shown that the NAMPT inhibitor FK866 reduces mucosal NAD^+^ levels and the activation of NF‐κB, then ameliorates DSS‐induced colitis.^[^ ^14^ ^]^ Moreover, clinical trials have been performed in advanced solid tumor malignancies to determine the toxicity profile and pharmacokinetics of FK866.^[^ ^56^ ^]^ However, it is unclear whether FK866 can alleviate the resistance of UC patients to IFX. Therefore, FK866‐mediated NAMPT blockade is a promising approach for treating resistance to IFX in patients with UC, further validation is needed.

In conclusion, our findings demonstrate that changes in the gut microbiota are associated with response to anti‐TNF therapy in patients with UC. These findings provide critical insights into the molecular mechanisms underlying the regulation of the NAD^+^ salvage pathway by *F. nucleatum* in the anti‐TNF responsiveness of UC. Interfering with the NAD^+^ salvage pathway or inhibiting the growth of *F. nucleatum* could alleviate the resistance of UC to anti‐TNF therapy, providing a new direction and target for the treatment of drug‐resistant UC.

## 4 Experimental Section

### Clinical Samples

All the included patients had a clinically and histologically confirmed diagnosis of UC. Fresh UC tissue samples (10 samples from RUC and 8 samples from NRUC) and normal tissue samples (5 cases) were collected from patients who underwent colonoscopy at the Gastrointestinal Endoscopy Center of the Second Affiliated Hospital of Chongqing Medical University. The fresh samples were rinsed three times with sterile physiological saline at 4 °C after collection and were stored at −80 °C for sequencing and RT‐PCR. Paraffin‐embedded UC intestinal tissues (89 samples) were collected from the pathology department of the same hospital, and the clinicopathological data of the patients were collected. RUC patients were defined as those with a Mayo score reduction of ≥ 3 points after 14 weeks of initial treatment with anti‐TNF (infliximab). NRUC patients were defined as those with a Mayo score reduction of ≤3 points after 14 weeks of initial infliximab treatment. Patients with clinical conditions requiring emergency management or involving malignancy, pregnancy, infectious diarrhea, or primary sclerosing cholangitis were excluded. Patients with a history of antibiotic drug use or fecal microbiota transplantation in the previous three months were excluded. Patients treated with other IBD‐related medications and those younger than 18 years of age were excluded from the NRUC and RUC groups. All participants have obtained informed consent, and the project was approved by the Medical Ethical Committee of the Second Affiliated Hospital of Chongqing Medical University (No. 806).

### Bacterial Strains, Cell Lines and Treatments

The *F. nucleatum* strain used in this study was isolated as described previously.^[^ ^8^ ^]^ The strain was stored at −80 °C in fetal bovine serum (FBS, Gibco, USA) and glycerol (50% v/v). *F. nucleatum* was incubated in fastidious anaerobe broth under anaerobic conditions at 37 °C in a shaker at 200–220 rpm min^−1^ for 3 to 4 d. The *Escherichia coli* strain DH5α (Tiangen, China) was incubated in Luria‐Bertani medium in a shaker at 200–220 rpm min^−1^ for 12 h at 37 °C. THP‐1 cells were cultured in RPMI 1640 medium supplemented with 1% penicillin‐streptomycin (Beyotime, China) and 10% FBS. The normal human epithelial cell line NCM460 and FHC cell line (ATCC, USA) were cultured in high‐glucose DMEM (Gibco, USA) supplemented with 10% FBS in 5% CO_2_ at 37 °C.

The cell lines were infected with *F. nucleatum* or *E. coli* at a multiplicity of infection of 1:100. 2% DSS (MP Biomedicals, USA) or 100 µg mL^−1^ IFX was added to the culture medium, and cells were incubated for 24 h. The FK866 inhibitor (Sigma, USA) was dissolved in dimethyl sulfoxide (DMSO; Thermo Fisher Scientific, USA), and the cells were treated with the FK866 inhibitor at the recommended concentration of 100 nm for 1 h before *F. nucleatum* infection.

### Animal Models and Experimental Design

All animal experiments were approved by the Animal Care and Use Committee of the Second Affiliated Hospital of Chongqing Medical University (approval number: #806). Three‐ to four‐week‐old male C57BL/6J mice were purchased from the Vital River Laboratory Animal Technology Company (Beijing, China), housed in a specific pathogen‐free environment with a natural light‐dark cycle, and provided with autoclaved food and water. All mice were acclimatized for 7 days and then randomized into the different treatment groups.

We performed FMT in DSS‐induced colitis mice to investigate the effect of the gut microbiota on the responsiveness to anti‐TNF therapy. The administration scheme is illustrated in Figure 1A. NRUC or RUC patients included in this study were randomly selected as fecal bacterial donors. Freshly collected donor fecal samples were immediately mixed in a biosafety cabinet under aerobic conditions, aliquoted into 2 mL freezing tubes, and stored at −80 °C. Freshly thawed human feces were resuspended in sterile phosphate‐buffered saline (PBS; 100 mg mL^−1^), filtered through a sterile 100 µm filter, vigorously shaken for 5 min, and centrifuged at 500 × *g* for 5 min, after which a fecal suspension was obtained.

The bacterial abundance was identified prior to gavage. Briefly, 200 µL of the fecal suspension at equal concentrations (1–3 × 10^8^ cells mL^−1^) were administered to mice via oral gavage. All mice (including those that did not receive FMT) were administered antibiotics, including 500 mg L^−1^ vancomycin, 200 mg kg^−1^ neomycin sulfate, 200 mg kg^−1^ metronidazole, and 200 mg kg^−1^ ampicillin, via gavage once a day for one week to establish an ABX mouse model. The mice were allowed to rest for 3 days after antibiotic treatment. FMT was performed via gavage with the fecal suspension (200 µL, 1–3 × 10^8^ cells mL^−1^) once a day for 2 weeks in the FMT groups. Moreover, the mice in the control and DSS groups were gavaged with 200 µL of a 0.9% saline solution. Three days after FMT completion, the mice received either normal drinking water or drinking water containing 3% DSS solution for 7 days. On day 3 after 3% DSS treatment, the mice were injected intraperitoneally with IFX (5 mg kg^−1^ twice a week) according to the group assignment.

To investigate the effect of *F. nucleatum* on the responsiveness to anti‐TNF therapy, we constructed a DSS‐induced colitis mouse model with *F. nucleatum* colonization. Briefly, on the third day after the ABX mouse model was established, mice were administered PBS containing *F. nucleatum* (10^9^ CFU mL^−1^) or PBS alone via daily gavage for 2 weeks. Three days after *F. nucleatum* infection, the mice received either normal drinking water or drinking water containing 3% DSS solution for 7 days. Depending on the group, the mice were administered FK866 (10 mg kg^−1^) intraperitoneally twice a day or the p38 MAPK‐specific inhibitor SB203580 (SB, MedChemExpress, USA) (5 mg kg^−1^) once a day, 60 min after DSS treatment. On day 3 after 3% DSS treatment, the mice were injected intraperitoneally with IFX (5 mg kg^−1^ twice a week) according to the group assignment.

Body weight, fecal characteristics, rectal bleeding, fecal occult blood, and the DAI were recorded daily during the modeling period. On day 10, the mice were sacrificed after 3% DSS treatment. Serum was collected via centrifugation (1000 × *g*, 15 min, 4 °C), and the colon length was measured. The distal colon was collected and fixed in 4% paraformaldehyde for FISH and HE staining. The remaining colonic samples were quickly frozen in liquid nitrogen and stored at −80 °C for subsequent western blotting and PCR analyses.

### DNA Extraction and High‐throughput Sequencing

A TIANamp Bacteria DNA Kit (Tiangen, China) was used according to the manufacturer's protocol to extract genomic DNA from frozen human clinical samples. The extracted DNA was sent to Shanghai OE Biotech Co., Ltd. (Shanghai, China) for sequencing. Agarose gel electrophoresis and a NanoDrop 2000 spectrophotometer (Thermo Fisher Scientific, USA) were used to measure DNA integrity and concentration, respectively. PCR amplification of the V3‐V4 variable regions of the bacterial 16S rRNA gene was carried out in 25 µL reactions using the universal primers 343F (5′‐TACGGRAGGCAGCAG‐3′) and 798R (5′‐AGGGTATCTAATCCT‐3′). Agencourt AMPure XP beads (Beckman Coulter Co., USA) were used to purify the PCR products, which were quantified using a Qubit dsDNA assay kit (Life Technologies, USA). After adjusting the PCR product concentration, sequencing was performed using an Illumina NovaSeq 6000 (Illumina Inc., San Diego, CA; OE Biotech Company; Shanghai, China). To detect and eliminate ambiguous bases (N), paired‐end reads were preprocessed using Trimmomatic software. Low‐quality sequences were removed using a sliding window trimming approach. FLASH software was used to assemble the paired‐end reads according to the following parameters: minimum overlap of 10 bp, maximum overlap of 200 bp, and maximum mismatch rate of 20%. QIIME software (version 1.8.0) was used to retain reads with 75% bases above Q20 after eliminating unsuitable reads, such as sequences smaller than 200 bp and homologous or ambiguous sequences. Clean reads with sequence similarities ≥97% were clustered into operational taxonomic units (OTUs) using VSEARCH software. The QIIME software package was used to select representative sequences for each OTU and all representative sequences were annotated against the Silva database (Version 132).

### Metabolomics Analysis

The analysis was performed with NCM460 cells co‐cultured with *F. nucleatum* or PBS (control). Ultra‐performance liquid chromatography and tandem mass spectrometry were used to analyze the sample extracts. Using a triple quadrupole linear ion trap mass spectrometer, each ion pair was scanned and detected based on the optimized declustering potential and collision energy. Mass spectrometry data were processed using Analyst 1.6.3 software. The metabolites in the samples were qualitatively and quantitatively analyzed using mass spectrometry data in local metabolic databases. Based on the retention time and peak type of each metabolite, the chromatographic peak of each metabolite detected in the different samples was corrected to ensure qualitative and quantitative accuracy. Principal component analysis was performed on the samples to determine the overall metabolic differences between the groups and the variability between the samples within the groups. Metabolite content data were normalized using unit variance scaling, and the accumulation patterns of metabolites among different samples were subjected to hierarchical cluster analysis using R software. Differential metabolites between groups were determined by a VIP ≥1 and fold change ≥2 or ≤ 0.5. The KEGG compound database was used to annotate the differential metabolites, and the annotated metabolites were mapped to the KEGG pathway database.

### Statistical Analyses

The paired or unpaired Student's *t*‐test and the Mann–Whitney U test were performed to compare the quantitative data between different groups. The relationship between *F. nucleatum* abundance and mRNA expression was analyzed by linear regression. The association between patient characteristics and *F. nucleatum* abundance was determined using Pearson's chi‐square test or Fisher's exact test. The differences between multiple groups were evaluated using one‐way analysis of variance. Categorical data were expressed as percentages. All *p* values were two‐tailed, and differences with a *p* value of <0.05 were considered significant (*p < 0.05, ** *p* < 0.01, and *** *p* < 0.001). Data were analyzed using SPSS Statistics software (version 26.0; IBM Inc., Chicago, Illinois, USA) and GraphPad Prism 8 software (GraphPad Software, Inc., San Diego, CA, USA).

## Conflict of Interest

The authors declare no conflict of interest.

## Supporting information



Supporting Information

Supporting Table 1

## Data Availability

The data that support the findings of this study are available in the supplementary material of this article.
