# Supplementary material for: The Gut Microbiota Affects Anti‐TNF Responsiveness by Activating the NAD+ Salvage Pathway in Ulcerative Colitis
Source: Adv Sci (Weinh). 2024 Dec 30;12(8):2413128. doi: 10.1002/advs.202413128 (PMC11848563; doi:10.1002/advs.202413128)

Supporting Information

**The Gut Microbiota Affects Anti-TNF Responsiveness by Activating the NAD^+^** **Salvage Pathway in Ulcerative Colitis**

*Jing Lei^#^, Lin Lv^#^, Li Zhong^#^, Feng Xu, Wenhao Su, Yan Chen, Zhixuan Wu, Song He***, Yongyu Chen**

**Supplementary Materials and Methods**

**Immunohistochemistry (IHC) and Western Blotting (WB)**

For IHC, paraffin-embedded tissue sections were stained using an UltraSensitive^TM^ SP (mouse/rabbit) IHC Kit (Maxim, China) according to the manufacturer’s instructions. Samples were evaluated by three pathologists who were blinded to the experimental protocols. The staining intensity (0-3) and the proportion of positive cells (0-4) were multiplied to obtain the composite expression score (ranges from 0 to 12), which in turn categorized the sections into low and high expression. For WB, total protein was extracted from cells or tissues with radioimmunoprecipitation assay (RIPA) lysis buffer mixed with 1% phenylmethanesulfonyl fluoride (PMSF). Total protein was separated via sodium dodecyl sulfate‒polyacrylamide gel electrophoresis (SDS-PAGE, 10% gel, Beyotime, China) and then transferred to polyvinylidene fluoride (PVDF) membranes (Millipore, USA). The membranes were blocked at room temperature for at least 2 h. After being washed three times with PBST, the membranes were incubated with primary antibodies overnight at 4 °C. After being washed three times with PBST, the membranes were incubated with secondary antibodies at room temperature for 1 h. Primary antibodies against the following targets were used: NAMPT (Proteintech, China), ZO-1 (Abcam, UK), Occludin (Abcam, UK), P38 (Cell Signaling Technology (CST), USA), phosphorylated-p38 (CST, USA), ERK (CST, USA), phosphorylated-ERK (CST, USA), JNK (Proteintech, China), phosphorylated-JNK (Proteintech, China), E-cadherin (CST, USA), vimentin (Proteintech, China) and GAPDH (Bioworld, USA).

**Enzyme-linked Immunosorbent Assay (ELISA)**

The blood was allowed to naturally coagulate at room temperature for 10-20 minutes and then was centrifuged at 2000-3000 rpm min^-1^ for approximately 20 minutes, after which the supernatant was collected. The concentrations of the targets were measured using mouse IL-1β, IL-6, IL-8, IL-10, IL-18, and TNF-α ELISA kits (Eβios, China) in accordance with the manufacturers' instructions.

**RNA Extraction and Real-Time PCR**

TRIpure Total RNA Extraction Reagent (ELK Biotechnology, China) was used to extract total RNA from NCM460 cells, and the PrimeScript™ RT reagent Kit with gDNA Eraser (TaKaRa, Japan) was used to synthesize the first strand cDNA. Quantitative real-time PCR was performed with EnTurbo™ SYBR Green PCR SuperMix Kit (ELK Biotechnology, EQ001) in triplicates on the QuantStudio 6 Flex System PCR instrument from Life Technologies. The 2^-ΔΔ^Ct method was used to compared the Ct values obtained from different samples. The internal reference transcripts were GAPDH and PGT. The primers used are displayed in supplementary material, Table S2.

**Isolation of Intestinal Epithelial Cells (IECs) from Colonic Tissue**

Colon tissue was isolated, and then the residual mesentery and mesenteric fat tissue was resected. After longitudinal sectioning, the colon tissues were rinsed with 4 °C PBS for 5 times to remove luminal contents including feces and mucus, and shredded into 1 mm^3^ pieces. Intestinal pieces were digested with 20 ml of DMEM supplemented with 300000 U L^-1^ collagenase Ⅺ and 0.1 g L^-1^ neutral protease Ⅰ and shaken horizontally at 180 rpm for 30 min at 37 °C. The suspensions were centrifuged at 1000 rpm min^-1^ for 5 min. The digestion buffer was discarded and the remaining pellets were resuspended in Percoll (GE Healthcare, USA) for 1 min and and centrifuged at 1500 rpm min^-1^ for 5 min. IECs were resuspended with culture medium and transfered to cell culture flask. The cells were incubated in 5% CO2 at 37 °C. The positive immunofluorescence for epithelial cell-specific cytokeratin 18 (Proteintech, China) was used to identify IECs.

**Cell Transfection**

siRNAs targeting the human NAMPT gene (siNAMPT) and nontargeting siRNAs were obtained from GeneChem (Shanghai, China). NCM460 cells were transfected with siRNAs according to the manufacturer’s instructions, and 3 replicate wells were set up for each transfected sample. Lipofectamine 6000 (Beyotime, China) was used for lipid-based transfections following the manufacturer’s protocol. The siRNA complex were incubated with cells the for 6 h and then replaced with fresh culture medium. Transfection efficiency was verified by western blotting after 3 days incubation.

**Fluorescence in Situ Hybridization (FISH)**

Five-micrometer-thick sections were prepared from samples obtained from pathology department archives and hybridized according to the manufacturer’s instructions (FOCOFISH, Guangzhou, China). 5’-CTT GTA GTT CCG C(C/T) TAC CTC-3’ was the sequence of *F. nucleatum*-targeted probe (FUS664; FITC labeled). 5’-GCT GCC TCC CGT AGG AGT-3’ was the sequence of the “universal bacterial” probe (EUB338; Cy3 labeled). Five 200 × magnification fields were randomly observed for each sample by three observers who were blinded to the experimental protocol, and the amount of bacterial fluorescence in each field was calculated. We defined the average number of visualized FUS664 probes per field as < 5, between 5 and 20, and > 20 as negative, low, or high abundance of *F. nucleatum*, respectively. The bacteria with more than 5 bacteria per field positive with the EUB338 probe but negative with the FUS664 probe were noted as other bacteria.

**Immunofluorescence Staining**

Paraffin sections were heated at 60 °C overnight and subsequently placed in xylene for 15 min. The sections were rehydrated in a descending concentrations of ethanol and washed in phosphate-buffered saline (PBS) for three times. Then the sections were immersed in sodium citrate buffer, boiled in a microwave oven for 8 minutes, and cooled to room temperature. After washed three times in PBS, the sections were incubated with goat serum for 10 min. Next, the sections were incubated with primary antibodies overnight at 4 °C. After washed three times in PBS, the sections were subsequently incubated with secondary antibodies at room temperature for 1 h. DAPI staining was used for nuclear staining.

**Supplementary Table**

Supplementary Table 2. List of Primers Used.

| Primers | Sequence5’-3’ | |
| --- | --- | --- |
| H-NRK1 | Forward | 5’- TTCGCCTTCACGCTCTATCTT-3’ |
|  | Reverse | 5’- CATCGAGGTGGCCTACAGTCT-3’ |
| H-NAPRT | Forward | 5’- CAACAACATTGACGAGGAGGC-3’ |
|  | Reverse | 5’- CATGTCCATGAGTGGAGACCC-3’ |
| H-GAPDH | Forward | 5’- CATCATCCCTGCCTCTACTGG-3’ |
|  | Reverse | 5’- GTGGGTGTCGCTGTTGAAGTC-3’ |
| H-NAMPT | Forward | 5’- TCGGTTCTGGTGGAGGTTTG-3’ |
|  | Reverse | 5’- TCCTATGTAAAGATAATCGGCCC-3’ |
| H-QAPRT | Forward | 5’- CCCTTGATTTCTCCCTCAAGC-3’ |
|  | Reverse | 5’- GTTGGGCTCAAGCTAGTGCC-3’ |
| H-NRK2 | Forward | 5’- TAGACACGCTGGAACAGGTTG-3’ |
|  | Reverse | 5’- GGTTTCATGATAGCAAGCGGG-3’ |
| M-IL-1β | Forward | 5’- GGGCCTCAAAGGAAAGAATCT-3’ |
|  | Reverse | 5’- GAGGTGCTGATGTACCAGTTGG-3’ |
| M-IL-6 | Forward | 5’- CTGGGAAATCGTGGAAATGAG-3’ |
|  | Reverse | 5’- AAGGACTCTGGCTTTGTCTTTCT-3’ |
| M-IL-10(M) | Forward | 5’- TACAGCCGGGAAGACAATAACT-3’ |
|  | Reverse | 5’- AGGAGTCGGTTAGCAGTATGTTG-3’ |
| M-TNF-α | Forward | 5’- TCCCCAAAGGGATGAGAAGTT-3’ |
|  | Reverse | 5’- GAGGAGGTTGACTTTCTCCTGG-3’ |
| M-GAPDH | Forward | 5’- TGAAGGGTGGAGCCAAAAG-3’ |
|  | Reverse | 5’- AGTCTTCTGGGTGGCAGTGAT-3’ |
| *F. nucleatum* | Forward | 5’-CAACCATTACTTTAACTCTACCATGTTCA-3’ |
|  | Reverse | 5’-GTTGACTTTACAGAAGGAGATTATGTAAAAATC-3’ |
| PGT | Forward | 5’-ATCCCCAAAGCACCTGGTTT-3’ |
|  | Reverse | 5’-AGAGGCCAAGATAGTCCTGGTAA-3’ |

**Supplementary Figures**

**Supplementary Figure 1.** Alpha diversity boxplot (ID: DSS+IFX, NID: NRUC+DSS+IFX, RID: RUC+DSS+IFX, NRID: NRUC+RUC+DSS+IFX).


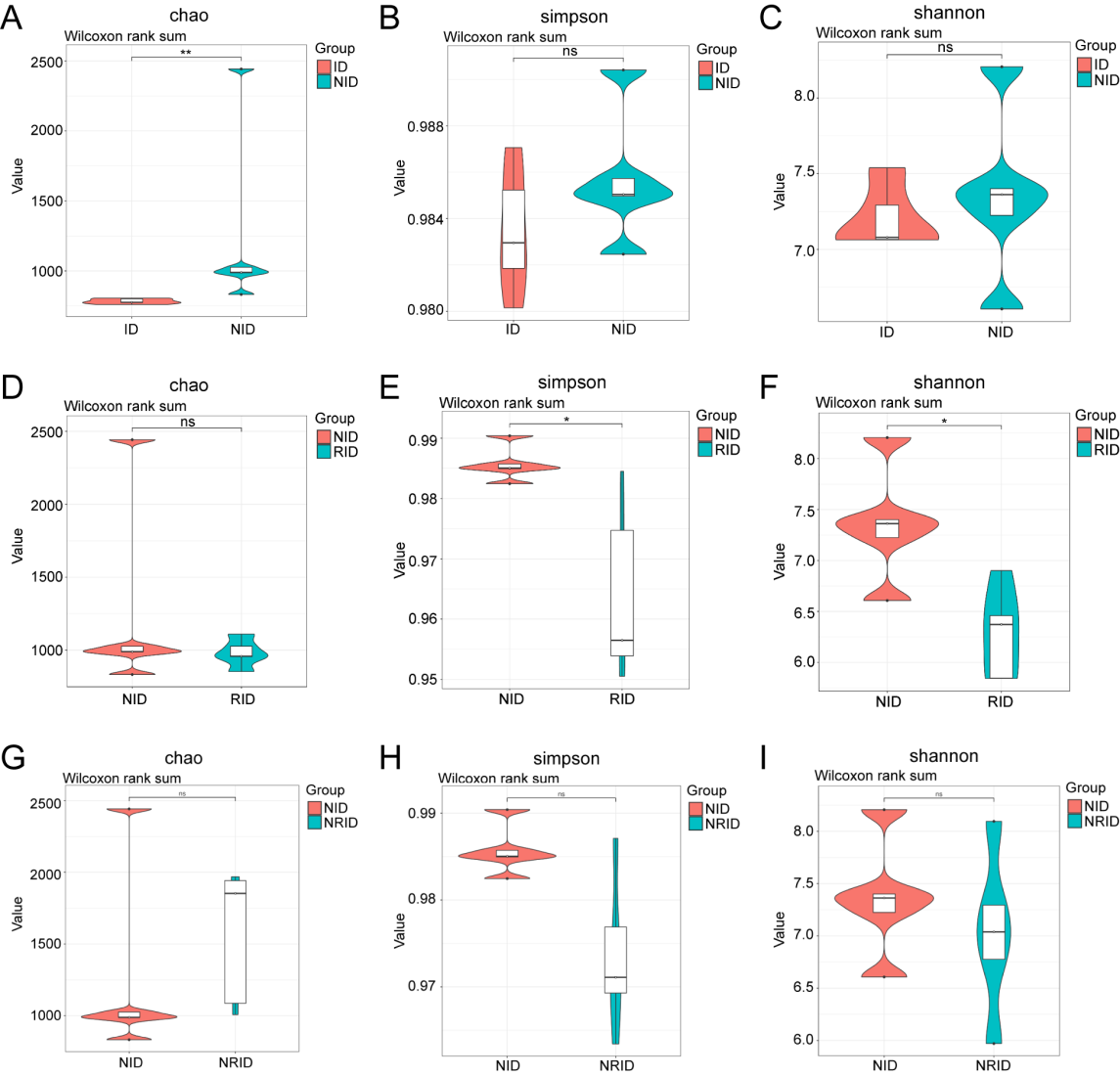


**Supplementary Figure 2.** Hierarchically clustered heat map analysis of the highly represented bacterial taxa (family level and genus level) in tissues from ID and NID mice (A, B), NID and RID (RUC+DSS+IFX) mice (C, D), RUC and NRUC patients (E, F) by 16S rDNA sequencing.


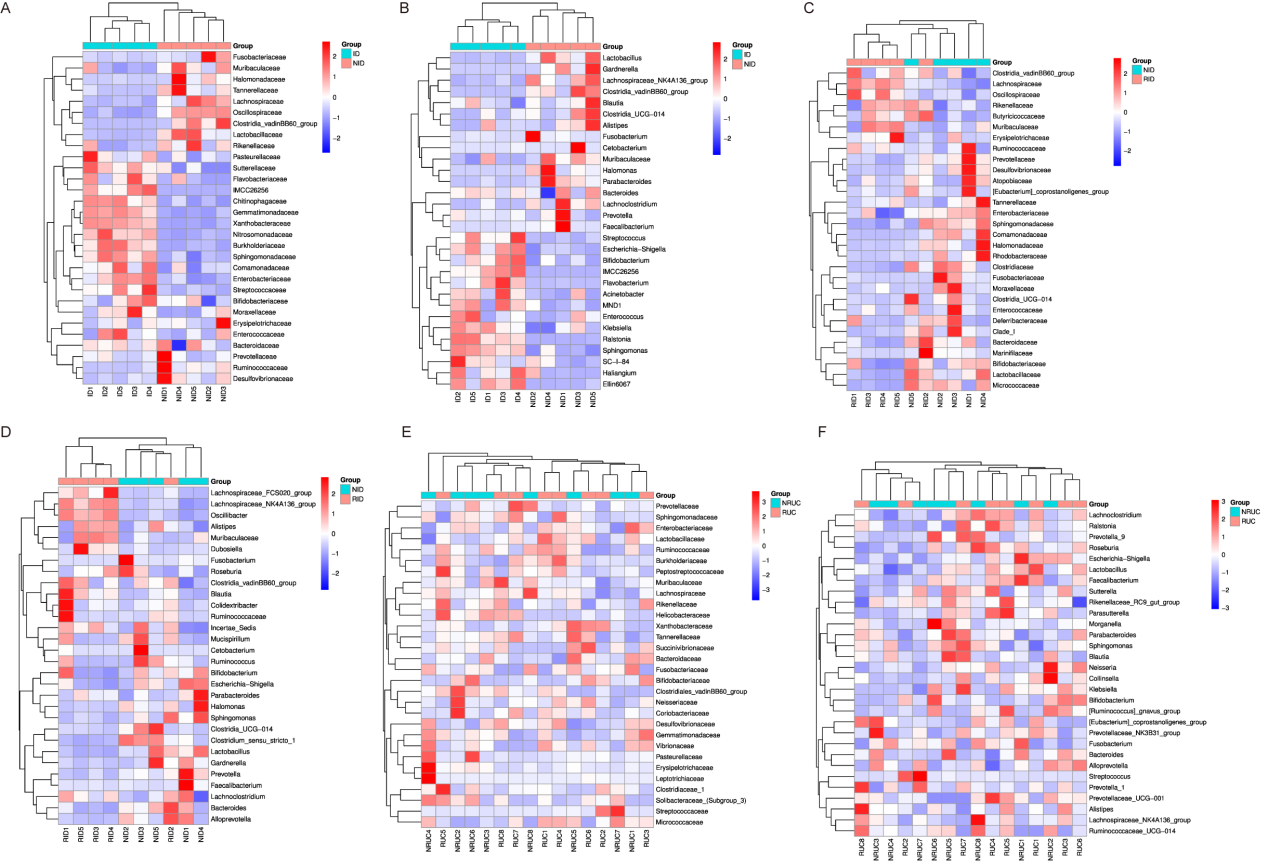


**Supplementary Figure 3.** Relative abundance of *F. nucleatum* in intestinal tissues (n = 5-6 per group; **P* < 0.05, ***P* < 0.01, and ****P* < 0.001; the dividing lines indicate any joins; nonparametric Mann-Whitney U test. Error bars indicate SD).


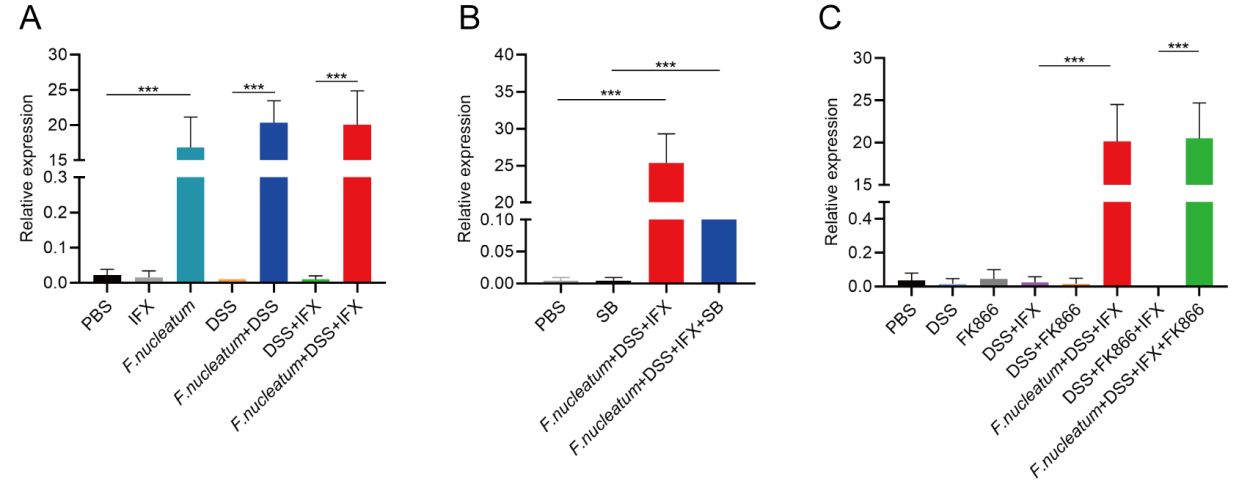


**Supplementary Figure 4.** Effects of *F.nucleatum* infection on intestinal immune cells.


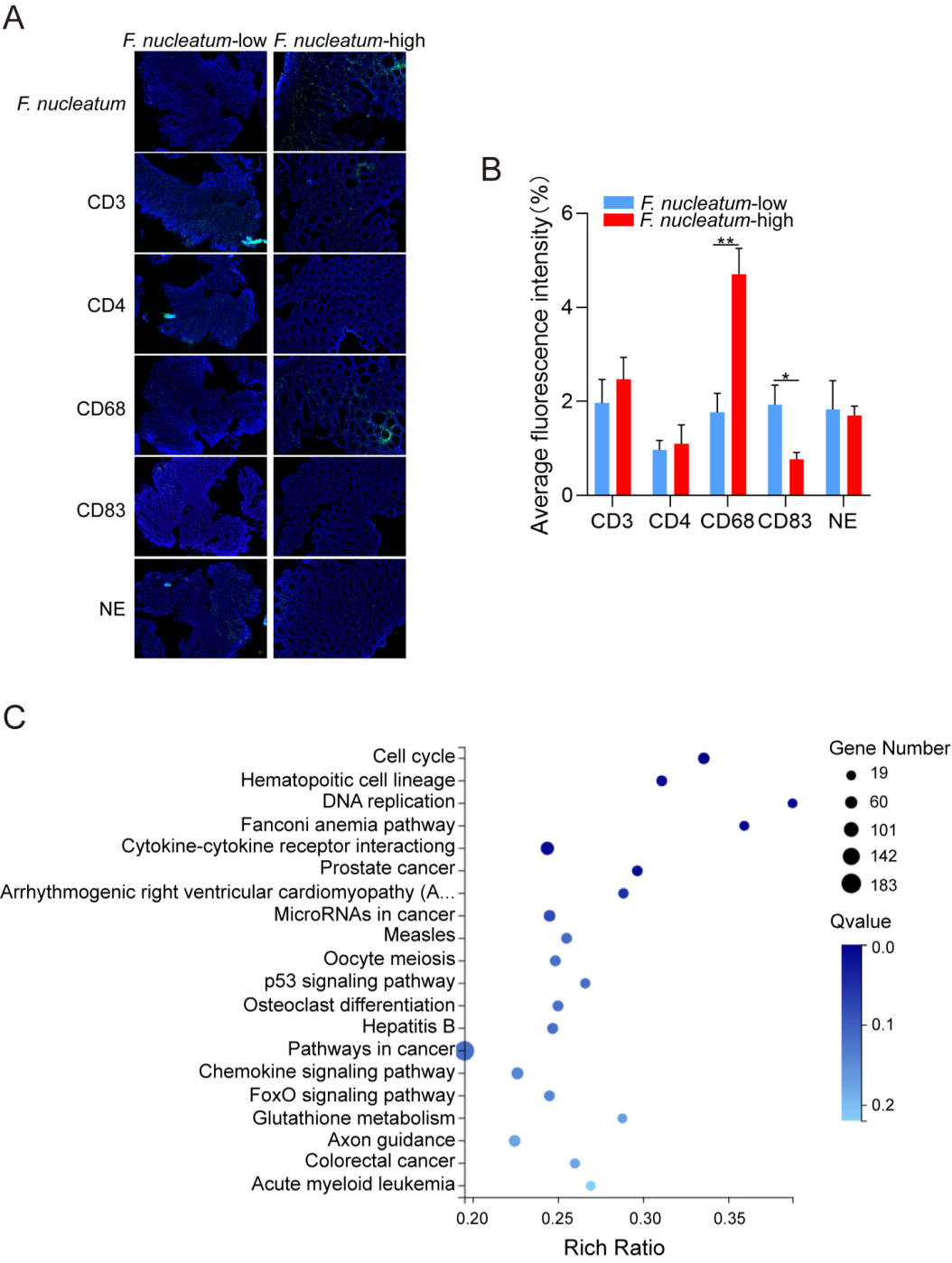


**Supplementary Figure 5.** Metabolomic analysis of NCM460 cells co-cultured with *F. nucleatum* or PBS (control).

**
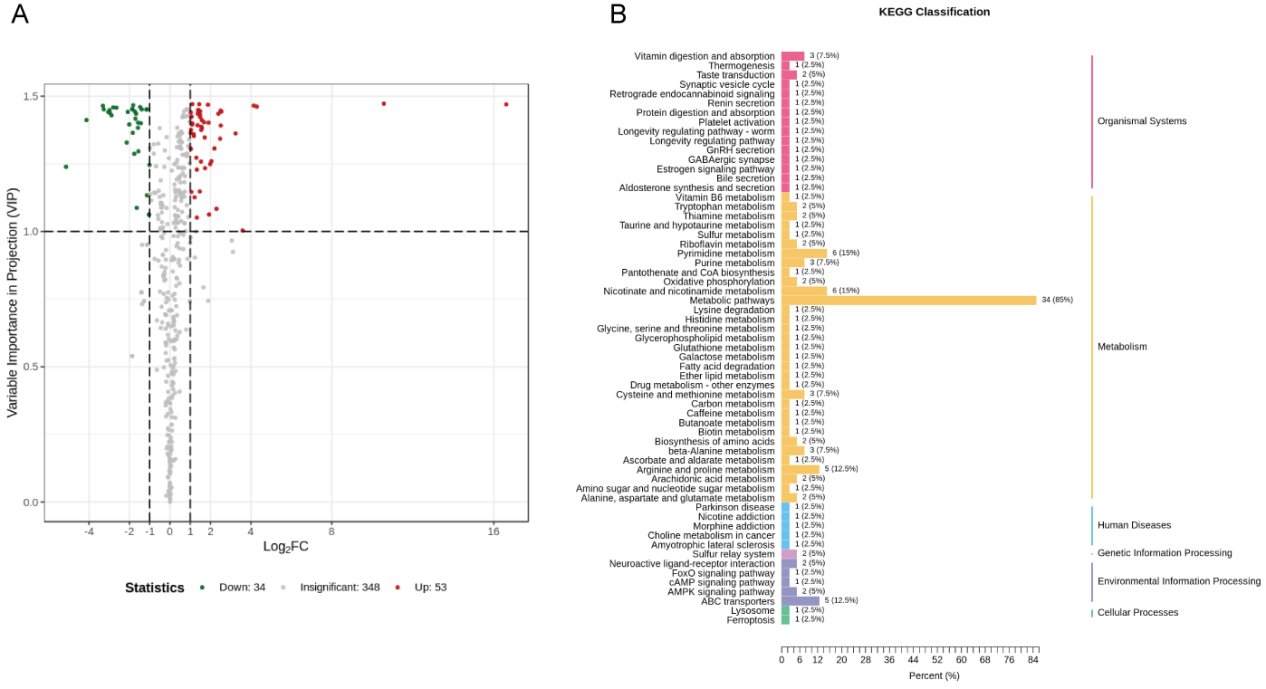
**

**Supplementary Figure 6.** Expression of inflammatory cytokines in mice serum and intestinal tissues (n= 5-6 per group; **P* < 0.05, ***P* < 0.01, and ****P* < 0.001; the dividing lines indicate any joins; nonparametric Mann-Whitney U test. Error bars indicate SD).


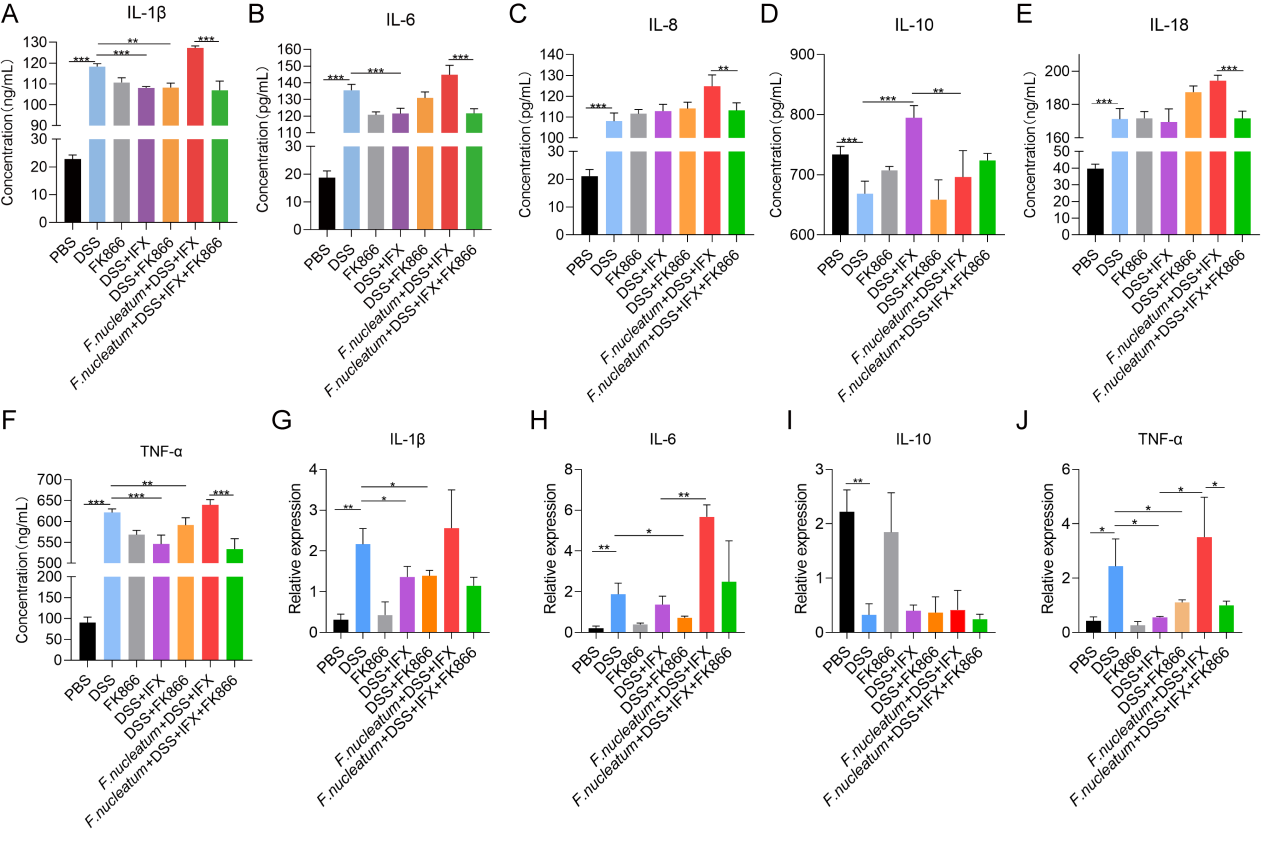

Supplement: Supplementary file 1 — Supporting Information [file ADVS-12-2413128-s002.docx]
